# Evaluation of the Position of Impacted Maxillary Canines on Panoramic Radiographs Using Deep Learning with CBCT as the Reference Standard

**DOI:** 10.3390/diagnostics16193167

**Published:** 2026-09-29

**Authors:** Anıl Demirel, Sabahattin Bor, Fırat Oğuz, Duygu Çelik Özen

**Affiliations:** 1 Department of Orthodontics, Faculty of Dentistry, İnönü University, Malatya 44280, Türkiye; venaroshan@gmail.com (S.B.); firat.oguz.3408@gmail.com (F.O.); 2 Department of Oral and Maxillofacial Radiology, Faculty of Dentistry, İnönü University, Malatya 44280, Türkiye; duygu.celik@inonu.edu.tr

**Keywords:** deep learning, impacted maxillary canine, position assessment, artificial intelligence, YOLOv11x-seg, panoramic radiography

## Abstract

**Background/Objectives:** This study aimed to detect impacted maxillary canines on panoramic radiographs using deep learning models, to classify their bucco-palatal position (buccal, mid-alveolar, palatal), to identify root resorption of adjacent teeth, and to classify the gamma angle according to the 65° threshold. **Methods:** A total of 683 panoramic radiographs containing impacted maxillary canines were retrospectively included, with cone-beam computed tomography used as the reference standard. For each of four predefined groups, the data were divided at the patient level into training (80%), validation (10%), and test (10%) sets, and an independent YOLOv11x-seg model was trained. **Results:** For impacted canine detection (Group I), the F1 score was 0.98. For positional classification (Group II), the highest performance was observed in the palatal position (F1 = 0.81; precision = 0.78; recall = 0.85), whereas lower performance was observed for the buccal and mid-alveolar positions (F1 = 0.59 for both). For root resorption (Group III), only one of nine resorption-positive teeth was correctly identified, resulting in an F1 score of 0.15 for the “resorption present” class and 0.73 for the “resorption absent” class. For gamma angle classification (Group IV), F1 scores were 0.70 above 65° and 0.77 below 65°. The critical success index values were 0.95, 0.51, 0.55, and 0.59 for Groups I–IV, respectively. **Conclusions:** The YOLOv11x-seg models showed promising performance for the detection and segmentation of impacted maxillary canines within the selected study population; however, their performance was limited for positional assessment and inadequate for reliable detection of root resorption on panoramic radiographs.

## 1. Introduction

Impacted teeth are defined as teeth that, despite the completion of the eruption age, fail to assume their normal position in the dental arch due to local and/or systemic factors and remain partially or completely embedded within the jawbones, with no expectation of eruption based on clinical and radiographic evaluations [1,2]. Maxillary canines are the second most frequently impacted teeth after third molars, with a reported prevalence ranging from 1% to 3% [3]. Numerous local and systemic factors contribute to tooth impaction [4]. It has been reported that buccally impacted canines are most commonly associated with arch length deficiency, whereas palatally impacted canines often present with sufficient eruption space [4]. Many investigators have indicated that impacted maxillary canines are more frequently located palatally than buccally [5,6].

The buccal, palatal, or mid-alveolar position of an impacted maxillary canine directly influences the surgical approach, orthodontic treatment planning, traction mechanics, and periodontal considerations; therefore, accurate determination of canine position is of significant clinical importance [7]. Due to the complexity of their management and the complications they may cause, early and accurate diagnosis of impacted maxillary canines is essential [8]. One of the most common complications is root resorption of adjacent teeth, particularly the lateral incisors [9,10]. The presence or absence of root resorption plays a fundamental role in determining the most appropriate treatment strategy [11]. Furthermore, the presence of root resorption directly affects the orthodontic treatment process. For these reasons, the present study aims not only to identify the position of impacted maxillary canines but also to enable the early and accurate detection of associated root resorption.

Clinical examination and radiographic methods are used diagnostically in the assessment of maxillary canines [12]. Supporting clinical findings with appropriate imaging modalities is essential for accurately determining the position of impacted canines and their relationships with adjacent teeth, which is critical for treatment planning and prognosis [12]. Periapical radiographs, panoramic radiography, and cone-beam computed tomography (CBCT) are commonly used in the diagnosis of impacted maxillary canines [8,13]. Among these modalities, panoramic radiography is the most widely used method due to its clinical accessibility, lower cost, and reduced radiation dose [8]. Although panoramic radiography is generally suitable for identifying the presence of an impacted maxillary canine, determining its precise buccopalatal position represents a more challenging diagnostic task. As a two-dimensional imaging modality, panoramic radiography cannot directly represent the buccolingual dimension, and anatomical superimposition may further limit positional assessment. Conventional two-dimensional radiographs present inherent diagnostic limitations in accurately localizing impacted canines and assessing adjacent root structures compared to three-dimensional imaging, where cone-beam computed tomography provides superior diagnostic accuracy for localization and treatment planning [14,15].

Although CBCT operates at a lower radiation dose than conventional computed tomography, it still exposes patients to higher levels of ionizing radiation compared with two-dimensional imaging techniques [16]. Increased radiation sensitivity, particularly in pediatric patients, necessitates careful consideration of CBCT use [17]. Due to disadvantages such as higher radiation dose, additional cost, and limited accessibility, maximizing the diagnostic information obtained from routinely acquired panoramic radiographs at the initial diagnostic stage may be clinically valuable. In this context, AI-assisted analysis may provide additional support for the initial assessment of impacted maxillary canines, without being intended to replace CBCT when three-dimensional evaluation is clinically indicated.

Because the evaluation of these images is time-consuming and prone to inter-clinician variability and potential errors, it is considered that integrating artificial intelligence–based methods to automate this process may provide significant advantages in terms of time efficiency and workload reduction during clinical decision-making. The use of artificial intelligence in medicine and dentistry has increased substantially in recent years [18], with AI-based approaches increasingly being investigated for diagnostic assessment, treatment planning, and clinical decision support in dental practice [19]. In particular, machine learning–based approaches such as convolutional neural networks have been widely applied across various fields of dentistry [20].

The application of artificial intelligence in orthodontics is relatively recent, with early studies and implementations primarily focusing on diagnostic and treatment processes [21]. Over the past decade, deep learning models have led to significant advances in dental image analysis. Among these developments, the You Only Look Once (YOLO) framework has emerged prominently in the literature by offering both high speed and accuracy for real-time object detection [22]. Prior to YOLO, object detection approaches typically required either running multiple classifiers on each image or employing a two-stage process in which candidate regions were first identified and subsequently classified [23]. In contrast, YOLO eliminated this multi-step workflow by adopting a single-stage, direct prediction approach, performing object detection through a single convolutional network [23].

YOLOv11, introduced by Ultralytics in 2024, builds upon previous versions and provides substantial improvements in accuracy, speed, and computational efficiency [24]. The model is available in several variants, including “n,” “s,” “m,” and “x,” among which the YOLOv11x variant has achieved the highest accuracy, outperforming all previous versions while maintaining remarkably low latency [24]. For these reasons, the YOLOv11x model was selected for use in the present study.

The primary aim of this study was to evaluate the ability of a deep learning–based artificial intelligence model to determine the position of impacted maxillary canines on panoramic radiographs. As secondary analyses, separate models were also evaluated for impacted canine detection, adjacent root resorption detection, and gamma angle classification according to the 65° threshold. CBCT images were used as the reference standard for impacted canine position and adjacent root resorption, whereas gamma angle measurements were performed on panoramic radiographs.

## 2. Materials and Methods

### 2.1. Patient Selection and Ethical Approval

The study was approved by the İnönü University Scientific Research Ethics Committee with decision dated 3 January 2025, and approval number 2025/6813. For the study material, a total of 9754 patient records of individuals who applied to the Department of Orthodontics, Faculty of Dentistry, İnönü University, for examination and/or treatment purposes between 2010 and 2025 were retrospectively reviewed. Among these patients, 1217 individuals with impacted maxillary canines were identified.

Radiographic records of patients with impacted maxillary canines were examined, and those who had both panoramic radiographs and cone-beam computed tomography (CBCT) images as part of their treatment process were selected. Panoramic radiographs and CBCT images were obtained during the pretreatment diagnostic process, with an interval ranging from approximately 1 week to 1 month, and no orthodontic treatment was performed between the two examinations. Following application of the inclusion and exclusion criteria, the eligible impacted canines comprised 352 buccal, 325 mid-alveolar, and 385 palatal cases. To enable the model to evaluate each positional category under equal conditions, the number of cases was balanced based on the least represented category. Accordingly, excess cases from the buccal and palatal categories were randomly excluded. Following this selection process, 534 radiographs were excluded, and the final study population consisted of 683 panoramic radiographs containing 975 impacted canines, with 325 canines in each positional category. The final study population consisted of 305 male and 378 female patients, with a mean age of 14 years and 8 months.

Participants were selected according to the following inclusion criteria:

- Individuals with both panoramic radiographs and CBCT images obtained for diagnostic purposes;
- Individuals without any craniofacial anomalies;
- Individuals with no history of orthodontic treatment;
- Individuals whose panoramic and CBCT images were of sufficient quality and suitable for evaluation;
- Individuals with impacted maxillary canines located in the mid-alveolar, buccal, or palatal region;
- Individuals with no history of dental trauma;
- Individuals aged 12 years and older [25,26].

Individuals meeting any of the following criteria were excluded from the study:

- Radiographic images containing metal artifacts or foreign objects within the field of view;
- Images with low diagnostic quality due to artifacts related to patient positioning or motion during image acquisition;
- Individuals with cleft lip and/or palate;
- Patients with severe root dilacerations or pathologies involving the impacted canine or adjacent teeth were also excluded from the study.

For all patients included in the study, CBCT images were acquired using the same device (NewTom 5G, QR Srl, Verona, Italy) with a field of view of 15 × 12 cm, an isotropic voxel size of 0.3 mm, a tube voltage of 110 kVp, and a tube current ranging 1–20 mA according to the automatic exposure control system. The total scan time was 18 s, with an exposure time of 3.6 s. Standard reconstruction was performed using an isotropic voxel size of 0.3 mm, with an axial slice thickness and axial pitch of 0.3 mm. Panoramic radiographs were obtained using a single panoramic unit (Planmeca Proline XC, Helsinki, Finland) at 66 kV and 5.0 mA, with an exposure time of 18 s and a pixel size of 0.08 × 0.08 mm. Study groups were established using panoramic radiographs that were free of artifacts, of adequate diagnostic quality, and acquired in accordance with standardized radiographic imaging protocols.

### 2.2. Evaluation and Labeling of Group I and Group II Radiographic Images

To determine the position of impacted maxillary canines, evaluations were performed using cone-beam computed tomography (CBCT) images. All CBCT images were recorded in Digital Imaging and Communications in Medicine (DICOM) format. All CBCT examinations were evaluated by a single oral and maxillofacial radiologist with 5 years of experience, who was blinded to the panoramic radiographic annotations and to the outputs of the deep learning models. DICOM images were examined in the sagittal, coronal, and axial planes using the manufacturer’s NNT software (version 16.4, QR Srl, Verona, Italy). The position of each impacted maxillary canine was classified as buccal, mid-alveolar, or palatal based on CBCT images, and the presence or absence of root resorption in the adjacent teeth was also assessed. Root resorption was diagnosed when a definite disruption of the external root contour with a resorptive defect extending into the dentin was observed on multiplanar CBCT images. Minor surface irregularities without clear dentinal involvement were not classified as root resorption. Root resorption was recorded as a binary variable (present/absent), and severity grading was not performed. For intraobserver reliability assessment, 30% of the CBCT examinations were randomly selected and reevaluated by the same radiologist after a five-week interval using the same diagnostic criteria. Intraobserver agreement was κ = 0.803 (87.5% agreement) for canine-position classification and κ = 0.833 (91.7% agreement) for root-resorption diagnosis.

**Figure 1:**
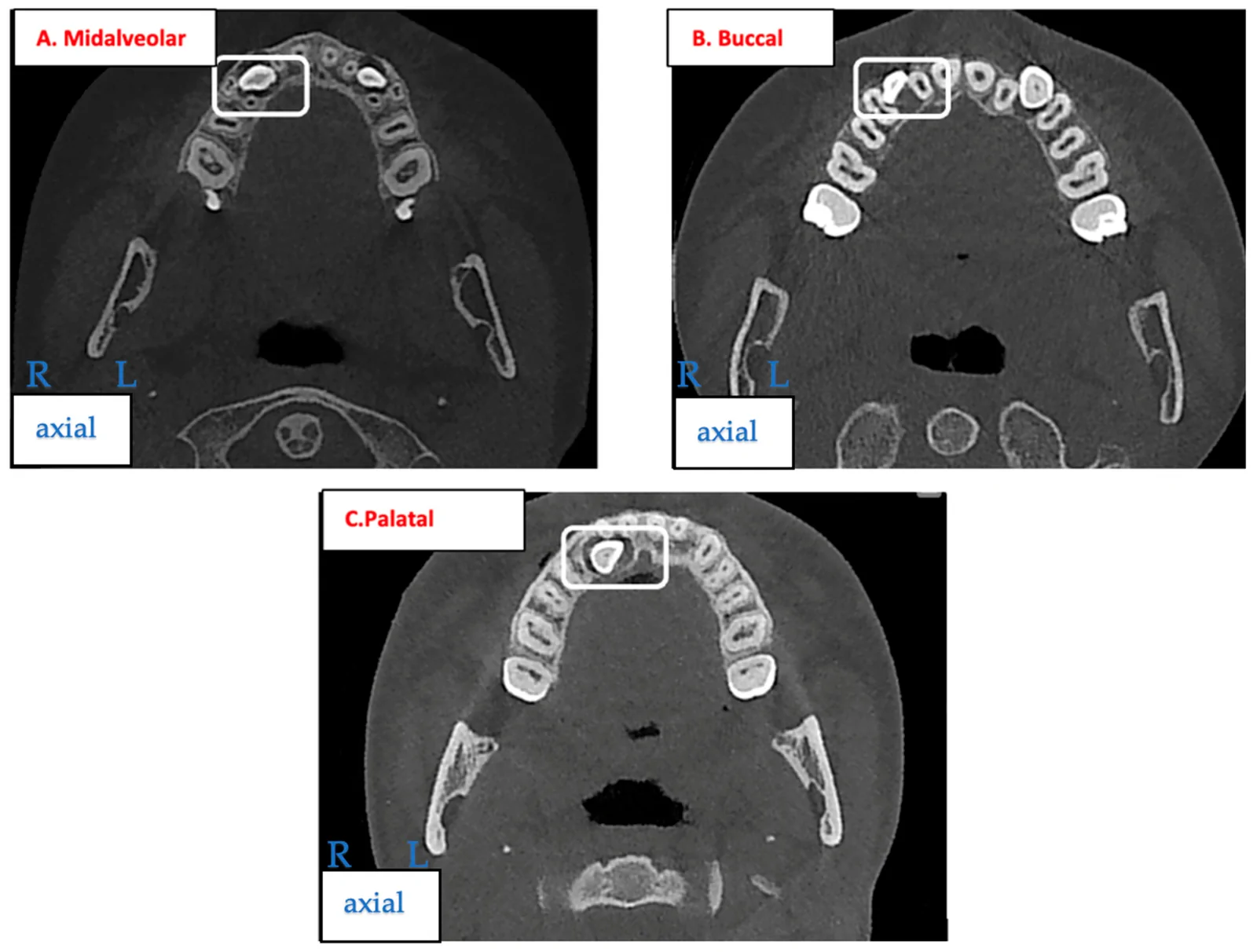
CBCT-based classification of impacted maxillary canines: (**A**) mid-alveolar, (**B**) buccal, (**C**) palatal. R, right; L, left.

DICOM images of 683 patients with impacted maxillary canines were examined three-dimensionally in sagittal, coronal, and axial sections. The position of each impacted maxillary canine was classified as buccal, mid-alveolar, or palatal (Figure 1). This evaluation was performed separately for a total of 975 maxillary canines, taking into account unilateral and bilateral impactions in individual patients, and all findings were recorded. Among these, six canines were identified as transposed, including two with lateral incisors and four with first premolars. Of these, three were classified as buccal, two as palatal, and one as mid-alveolar in Group II.

During the analysis process, to ensure statistical balance and to improve the learning performance of the model, 325 samples from each positional category were selected to form three equally sized groups. Thus, data balance was achieved among the buccal, mid-alveolar, and palatal position groups.

The process of marking and classifying the locations of objects within an image is referred to as labeling (annotation). The panoramic radiographs included in this study were uploaded to the two-dimensional labeling module of the CranioCatch software (Version 1, Eskişehir, Türkiye), and a separate project was created for each study group.

**Figure 2:**
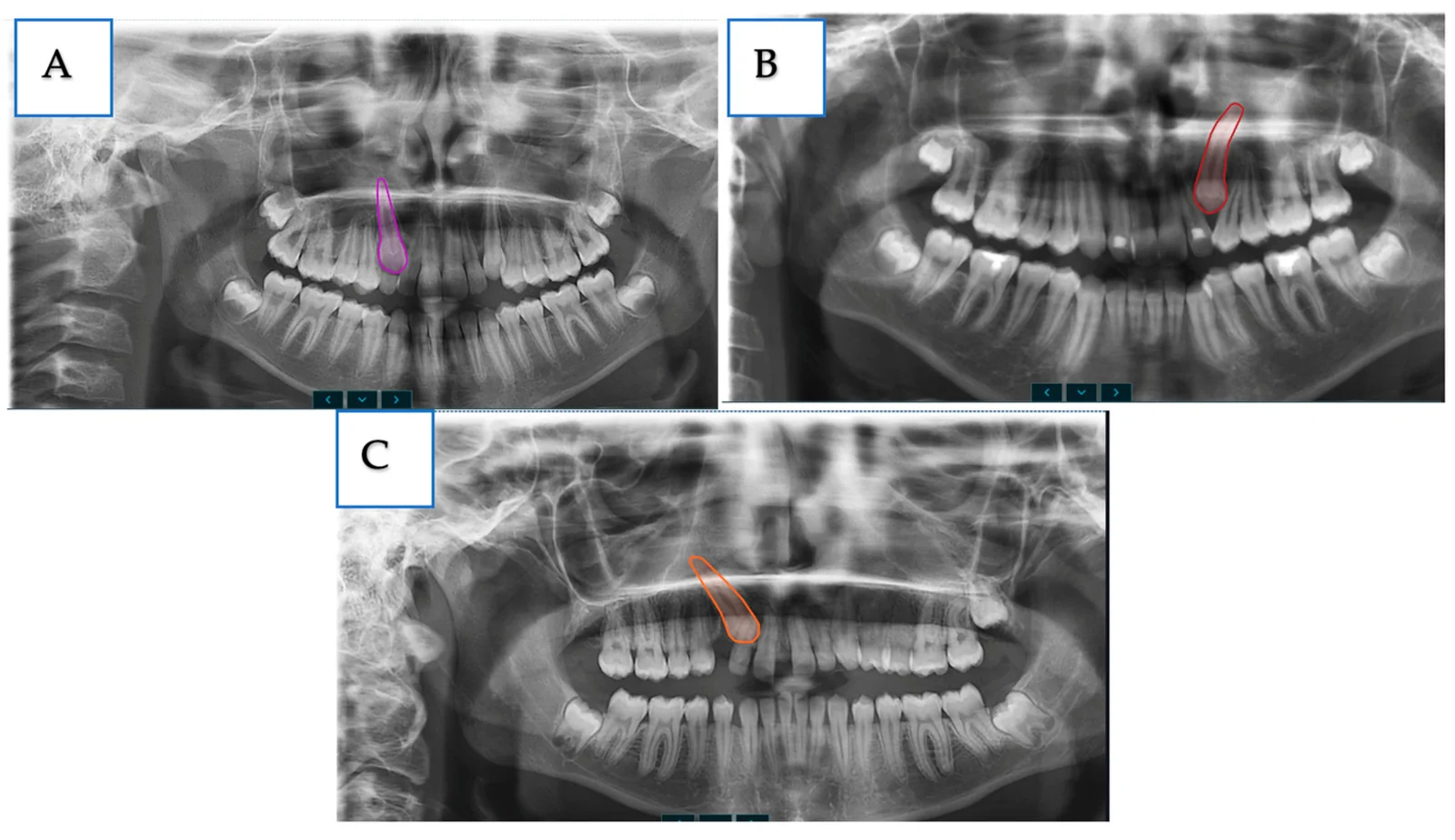
Labeling of impacted maxillary canines on panoramic radiographs: (**A**) mid-alveolar, (**B**) buccal, (**C**) palatal.

For Group II, manual labeling was performed on a total of 975 impacted maxillary canines identified on 683 panoramic radiographs uploaded to the system, with preservation of the tooth morphology. Following the labeling process, the corresponding positional category of each labeled tooth was selected. Representative examples of labeling according to the three different positional categories for this group are presented in Figure 2.

In Group I, the same 975 labels derived from the 683 panoramic radiographs used in Group II were utilized to define and localize impacted maxillary canines, and no additional labeling procedure was performed. All radiographs in Group I were obtained from patients with at least one impacted maxillary canine; therefore, no radiographs from patients without impacted maxillary canines were included. For all study groups, the labeling process was performed by a research assistant (AD). All labels were subsequently reviewed by two orthodontic specialists (FO and SB) to verify the accuracy of tooth boundaries and anatomical consistency. Any annotations requiring modification were revised based on the specialists’ assessment before being finalized for model training and evaluation.

### 2.3. Evaluation and Labeling of Group III Radiographic Images

**Figure 3:**
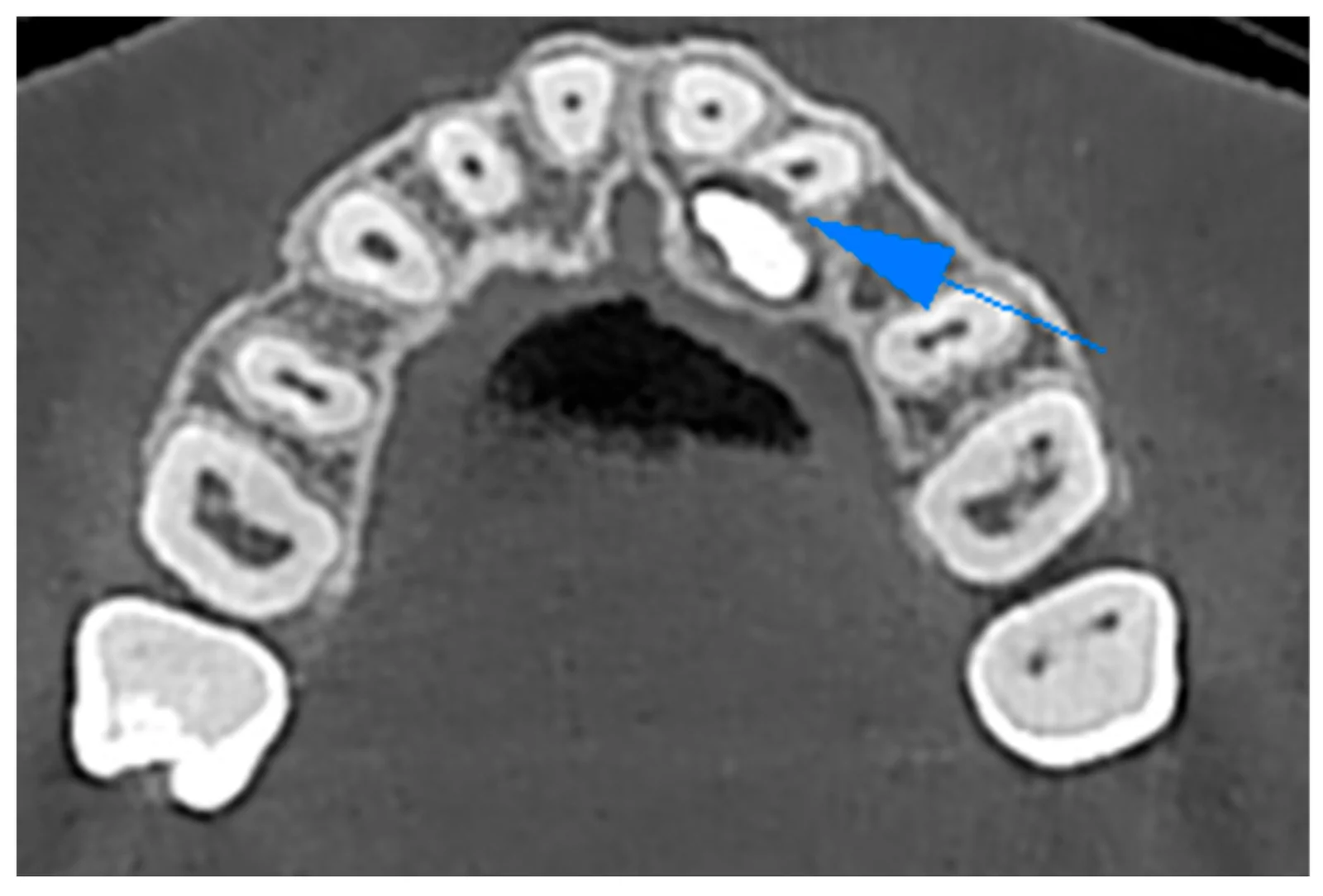
CBCT image demonstrating root resorption of a lateral incisor caused by an impacted maxillary canine. The arrow indicates the area of root resorption.

In Group III, DICOM images of 510 patients with impacted maxillary canines were examined three-dimensionally in collaboration with an Oral and Maxillofacial Radiology specialist to assess possible root resorption in teeth adjacent to the impacted canines. The remaining 173 patients were excluded from Group III due to insufficient panoramic image quality or inadequate visualization of the adjacent teeth for reliable labeling; this exclusion was independent of root resorption status. Following the evaluation, the presence of root resorption in the roots of adjacent teeth was determined, and the findings were recorded (Figure 3).

Among the 1312 evaluated incisor and premolar teeth, root resorption was identified in a total of 90 teeth. Root resorptions not associated with impacted canines were excluded from the analysis. Adjacent incisors and premolars were classified into two subcategories: “resorption present” and “resorption absent.”

**Figure 4:**
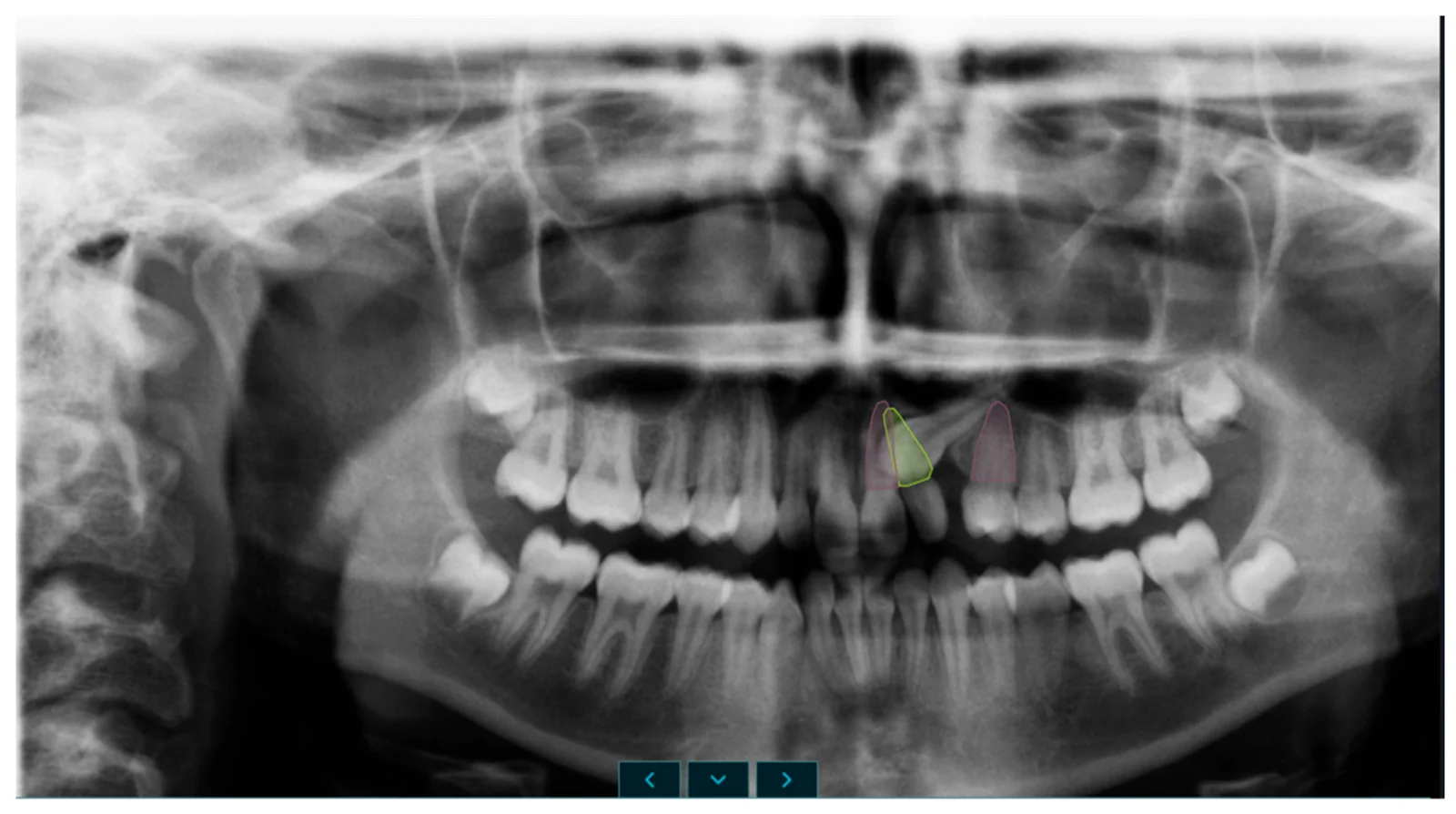
Labeling of root resorption in teeth adjacent to impacted maxillary canines in Group III.

In 510 panoramic radiographs, the roots of 1312 teeth located adjacent to impacted maxillary canines were manually labeled while preserving root morphology. Following the labeling process, the resorption status of each tooth root was designated as either “resorption present” or “resorption absent.” Representative labeling images for this group are shown in Figure 4.

### 2.4. Evaluation and Labeling of Group IV Radiographic Images

**Figure 5:**
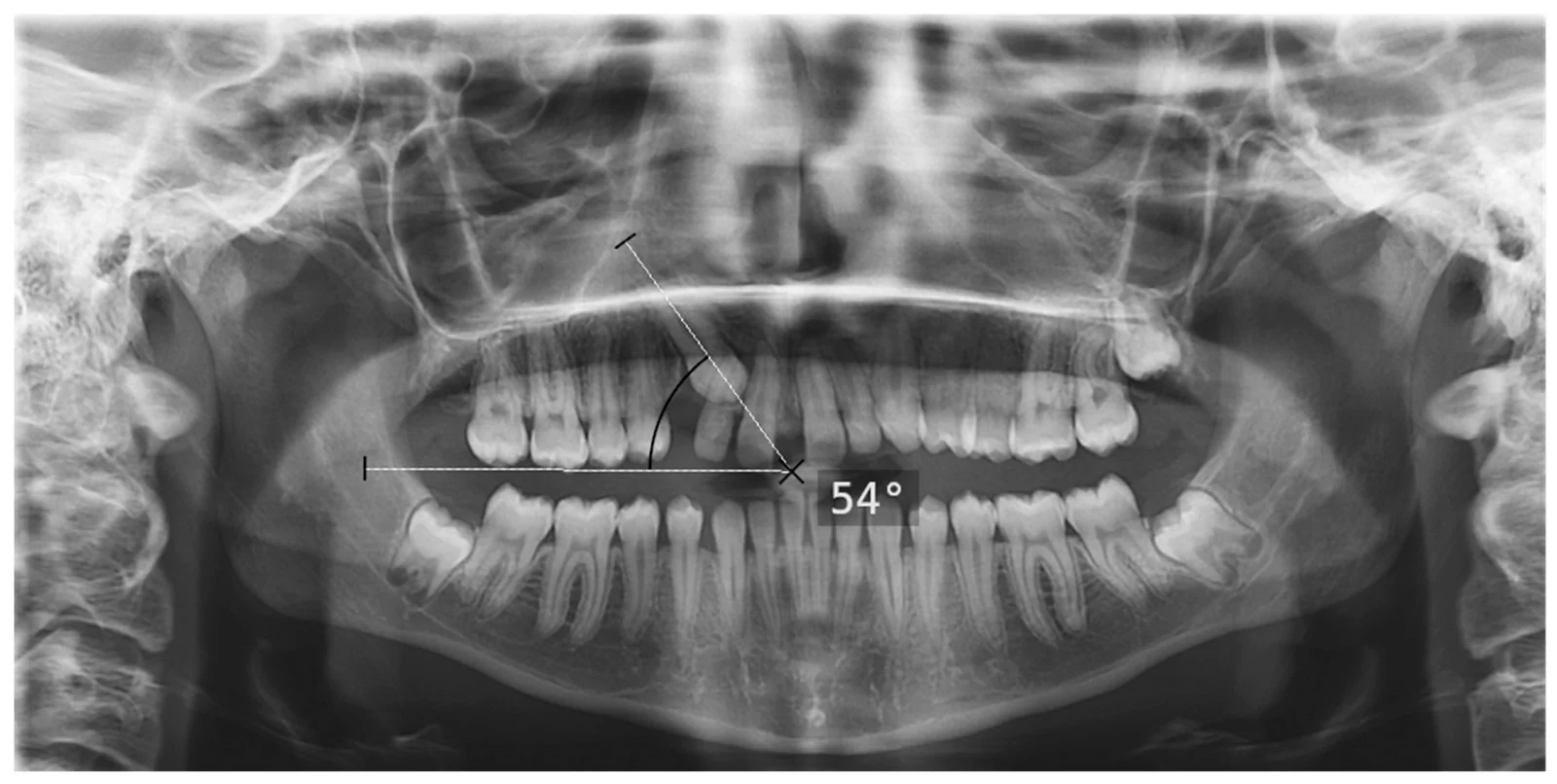
Measurement of the angle between the long axis of the maxillary canine and the occlusal plane on panoramic radiographs.

In Group IV, panoramic radiographs of 500 patients were imported into Blender software (v4.3.2, Blender Foundation, Amsterdam, The Netherlands) for gamma angle measurement. Blender was selected because its measurement tools enabled standardized definition of the canine long axis and occlusal plane directly on panoramic radiographs and measurement of the angle between these reference lines within a consistent workflow. In addition, Blender is freely available and readily accessible. The gamma angle was defined as the angle between the long axis of the maxillary canine and the occlusal plane [27,28]. Using Blender software, the gamma angle for each maxillary canine was manually measured and calculated, and the obtained values were recorded (Figure 5). Intraobserver reliability was assessed by repeating the measurements on 63 randomly selected impacted canines after a one-month interval by the same researcher (A.D.). Interobserver reliability was evaluated by having 25 randomly selected impacted canines independently measured by a second researcher (F.O.). All obtained data were recorded in Excel software (Microsoft Office 365, Redmond, WA, USA). For Group IV, the reference labels were derived from these manual gamma angle measurements performed on panoramic radiographs and were not based on CBCT. The measured gamma angles were categorized according to the 65° threshold, and these categories were used as the ground-truth labels for binary classification.

A total of 648 maxillary canines were manually labeled while preserving tooth morphology. Two canines were excluded from Group IV because the occlusal plane could not be clearly identified on the panoramic radiographs, preventing reliable gamma angle measurement. Subsequently, the corresponding angular value for each labeled tooth was assigned. The measured angles were categorized into two groups, below 65° and above 65°, based on the reference values reported by Katsnelson et al. [28]. Since Katsnelson et al. [28] evaluated only buccal and palatal positioned canines, maxillary canines located in the mid-alveolar position were excluded from this grouping in the present study.

### 2.5. Training of the Deep Learning Models and Dataset Construction

In this study, four independent YOLOv11x-seg models based on the YOLOv11 architecture were used, implemented using the open-source Python programming language and the PyTorch deep learning library (Python 3.8; Ultralytics 8.3.123). The model training process was conducted in a computing environment equipped with an NVIDIA Tesla V100 graphics processing unit with 16 GB of memory. The YOLOv11x-seg models were initialized with COCO pre-trained weights and subsequently fine-tuned on the study dataset. The standard YOLOv11x-seg architecture was used without modification; it consists of a backbone for feature extraction (convolutional, C3k2, SPPF, and C2PSA blocks), a neck for multi-scale feature aggregation, and a segmentation head that predicts the bounding box, class, and instance mask of each object [24].

For each study group, the data were randomly divided into three subsets consisting of 80 percent training, 10 percent validation, and 10 percent test data. Data splitting was performed at the patient level, ensuring that all data belonging to the same patient were assigned exclusively to a single subset and did not appear across the training, validation, and test sets. Panoramic radiographic images for each group were resized to 1280 × 1280 pixels. Each model was trained using the YOLOv11x-seg architecture with a batch size of 2 and a maximum training limit of 800 epochs. The optimizer was automatically selected using the Ultralytics training configuration (optimizer = auto), which selected stochastic gradient descent (SGD). The initial learning rate (lr0) was set to 0.01 and was dynamically adjusted during training using the Ultralytics learning-rate scheduler (linear decay; cosine scheduling was not used). A warm-up period of 3 epochs (warm-up momentum, 0.8; warm-up bias learning rate, 0.1) was applied, and the final learning-rate factor (lrf) was set to 0.01. Early stopping with a patience of 50 epochs was implemented to reduce the risk of overfitting. Therefore, 800 epochs represented the predefined maximum training limit rather than a fixed number of training epochs. The final model checkpoint was selected based on the best performance on the validation dataset. Momentum and weight decay were set to 0.937 and 0.0005, respectively. A random seed of 0 was used, with deterministic training enabled. Automatic mixed precision was used during training. The loss function comprised bounding-box regression, classification, distribution focal, and segmentation mask components, with loss gains of 7.5 (box), 0.5 (classification), and 1.5 (distribution focal). The IoU threshold for non-maximum suppression was set to 0.7, and the maximum number of detections per image was 300. No manually fixed confidence threshold was specified in the training configuration. Data augmentation included HSV hue (0.015), saturation (0.7), and value (0.4) adjustments, translation (0.1), scaling (0.5), horizontal flipping (*p* = 0.5), and mosaic augmentation (disabled during the final 10 epochs); rotation, shear, perspective transformation, and vertical flipping were not applied. The following additional augmentations were used: Blur (*p* = 0.05), CLAHE (*p* = 0.05), GaussNoise (*p* = 0.05), ISONoise (*p* = 0.10), MultiplicativeNoise (*p* = 0.10), RandomBrightnessContrast (*p* = 0.05), RandomSnow (*p* = 0.05), Sharpen (*p* = 0.05), and ToSepia (*p* = 0.005). The training and validation loss curves for the four independently trained models are provided in Supplementary Figure S1.

**Figure 6:**
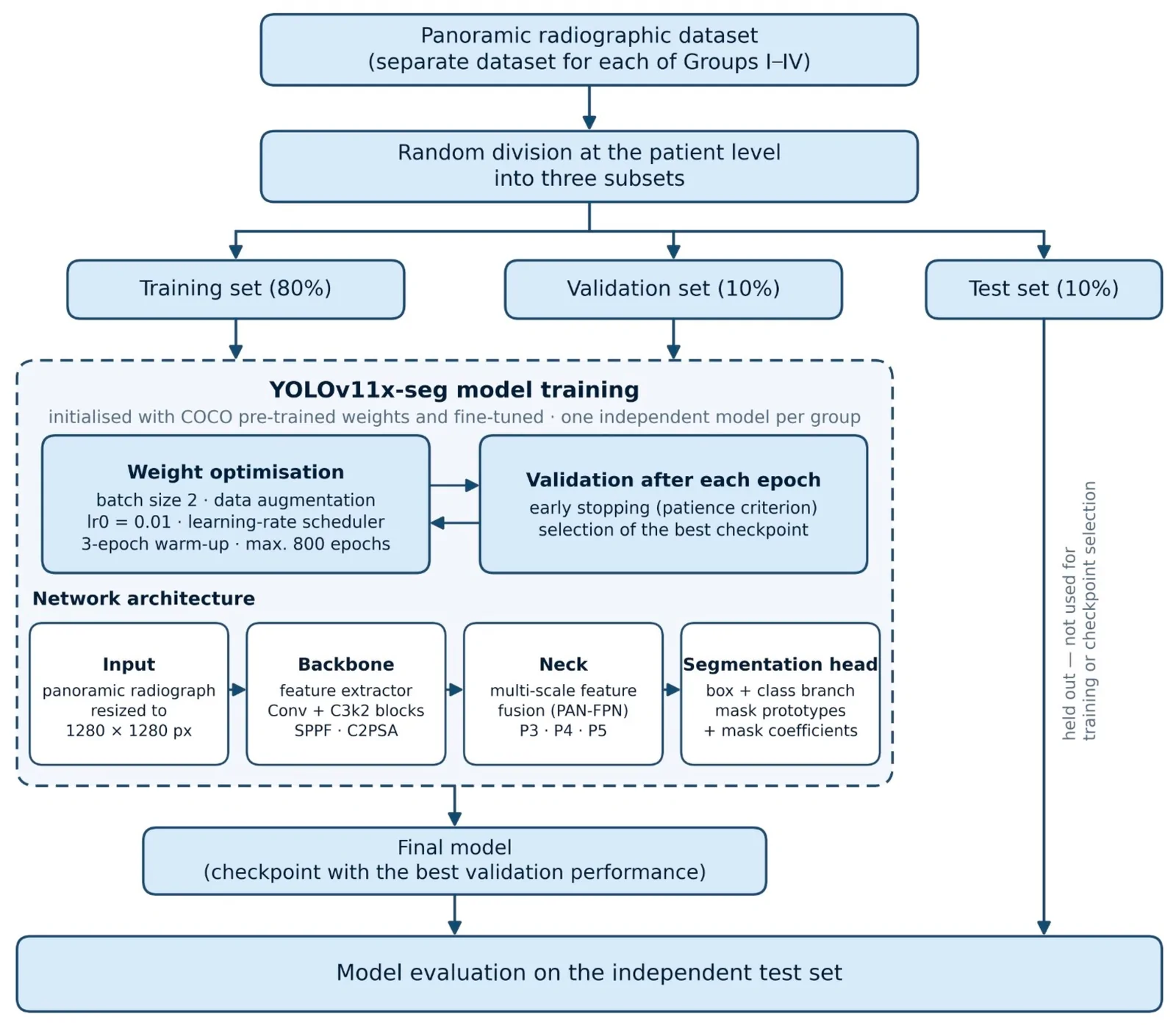
Schematic diagram of the YOLOv11x-seg model-training framework applied independently to Groups I–IV.

The overall schematic diagram of the YOLOv11x-seg model-training framework applied independently to the four study groups is presented in Figure 6.

**Figure 7:**
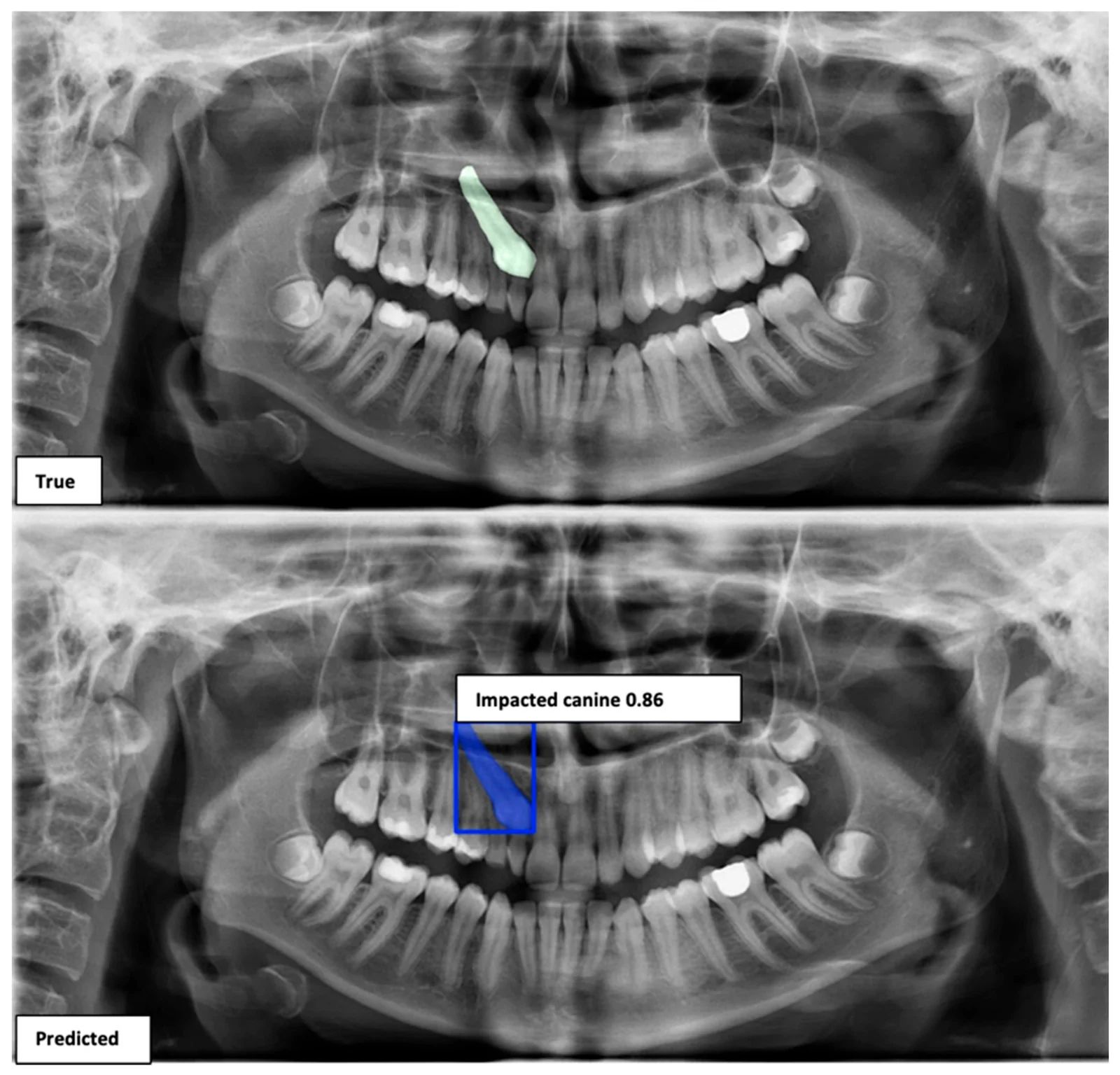
Ground truth annotations and YOLOv11x-seg predictions for Group I.

In Group I, a comparative visualization of the ground truth labels and the predictions generated by the deep learning model on panoramic radiographs is presented in Figure 7.

### 2.6. Parameters Used to Evaluate the Performance of the Deep Learning Models

A confusion matrix is used to evaluate the performance of the developed deep learning models based on their test results. This matrix summarizes the performance of the artificial intelligence algorithm in tabular form by comparing the true class labels with the model predictions [29]. In the confusion matrix used in this study, columns represent the true values, while rows represent the predicted values generated by the deep learning model.

To evaluate the performance of the deep learning model, the critical success index, precision, recall, and F1 score were calculated and used as performance metrics. These measures were derived using true positive, false positive, and false negative values, because true negatives are not defined in an object detection/instance segmentation task. Ninety-five percent confidence intervals (CIs) for precision and recall were calculated as exact (Clopper–Pearson) binomial intervals from the confusion-matrix counts, and those for the F1 score, critical success index, and the macro- and micro-averaged values were obtained by nonparametric bootstrap resampling of the confusion-matrix observations (100,000 resamples, percentile method).

Within the scope of model performance evaluation, a Receiver Operating Characteristic (ROC) curve was generated, and the Area Under the Curve (AUC) value was calculated. In addition, the Jaccard index (Intersection over Union), Dice coefficient, mAP at 0.5, and mAP at 0.5 to 0.95 were also computed.

### 2.7. Performance Evaluation Metrics

Critical success index: Because true negatives are not defined in an object detection/instance segmentation task, overall performance was summarized using the critical success index (CSI; also known as the threat score), calculated as TP/(TP + FP + FN). The TP, FP, and FN values were determined separately for each evaluated class and then aggregated across classes, and the background entries of the confusion matrix were included in the FP and FN counts. Because true negatives do not contribute to this index, it is a stricter measure than conventional accuracy; it is calculated at the instance level and is distinct from the pixel-level Jaccard/Intersection over Union value used to evaluate the segmentation masks.

Precision: Precision represents the proportion of correctly predicted positive samples among all samples predicted as positive by the model [30].

Recall (Sensitivity): Recall measures the ability of the model to correctly identify true positive samples for each class and reflects the effectiveness of the model in detecting positive cases [30].

F1 Score: The F1 score is the harmonic mean of precision and recall. Higher F1 score values indicate better classification performance [31]. It is considered a particularly useful metric for evaluating model performance in imbalanced datasets.

Receiver Operating Characteristic (ROC) Curve: The ROC curve evaluates the performance of a classification model at different threshold values by illustrating the relationship between the true positive rate and the false positive rate.

Area Under the ROC Curve (AUC): The AUC value is obtained by calculating the area under the ROC curve and ranges between 0 and 1. Higher AUC values indicate a better ability of the model to discriminate between classes [32].

In the present study, ROC analysis was performed separately for each class. Ground-truth polygon annotations were converted into binary masks, with pixels belonging to the corresponding class assigned a value of 1 and background and other-class pixels assigned a value of 0. Each predicted segmentation mask was multiplied by the confidence score of the corresponding prediction. When multiple predictions of the same class were present, the highest score at each pixel was retained. The ground-truth masks and prediction score maps were then flattened and pooled into a single array across all test images for each class. Because prediction scores at multiple thresholds were not available, the true positive rate (TPR; recall/sensitivity) and false positive rate (FPR) were calculated at the model’s default confidence threshold, and the AUC was derived from the single operating point defined by these pixel-level TPR and FPR values (i.e., the area under the curve connecting (0, 0), the operating point, and (1, 1)). The AUC values are therefore descriptive and do not represent a threshold-independent ROC analysis; for the same reason, confidence intervals could not be calculated for them.

Intersection over Union (IoU) or Jaccard Index: IoU is defined as the ratio of the intersection area between the predicted segment and the ground truth segment to the union of these two segments. The mAP at 0.5 represents the mean Average Precision across all classes when the IoU threshold is set to 0.5, whereas mAP at 0.5 to 0.95 indicates the average of AP values calculated across multiple IoU thresholds ranging from 0.5 to 0.95 with increments of 0.05 [33].

Dice Coefficient: The Dice coefficient is a similarity metric that measures the degree of overlap between the predicted segmentation and the ground truth labels. It ranges from 0 to 1, with values closer to 1 indicating greater agreement between the two regions.

## 3. Results

### 3.1. Results Obtained from the Group I Test Dataset

The performance metrics obtained for the detection of impacted maxillary canines in the Group I test dataset are presented in Table 1. The critical success index, precision, recall, and F1 score achieved from the Group I test dataset were 0.95, 0.97, 0.98, and 0.98, respectively. In addition, the mean Average Precision values of the model were mAP at 0.5 of 0.99 and mAP at 0.5 to 0.95 of 0.83. The Dice coefficient, reflecting segmentation performance, was calculated as 0.91, while the Jaccard or Intersection over Union value was 0.85.

Among the six transposed canines identified in the entire dataset (two involving the lateral incisors and four involving the first premolars), one was included in the independent test set and was correctly detected and localized by the model.

**Figure 8:**
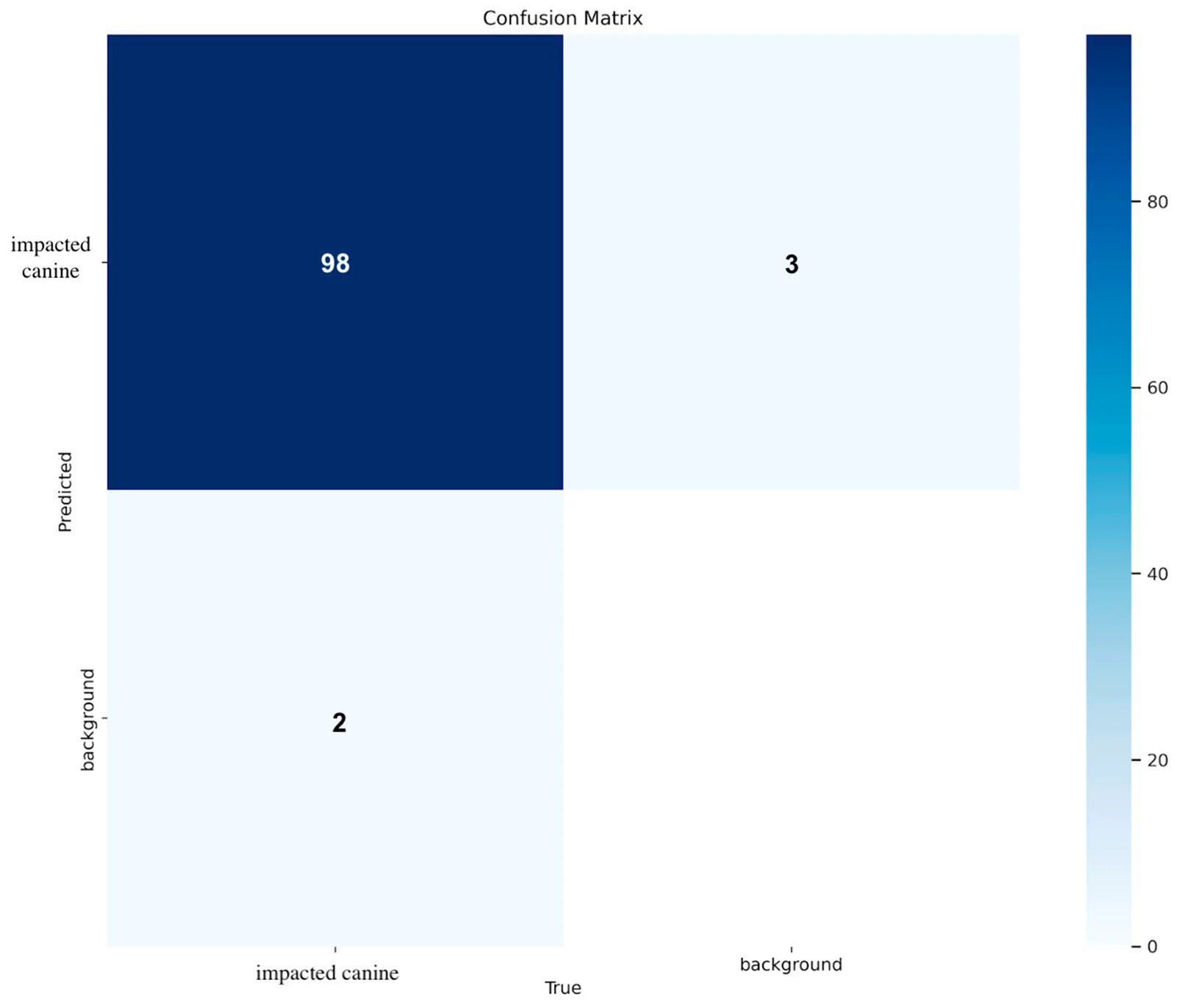
Confusion matrix for the Group I test dataset.

Figure 8 illustrates the confusion matrix of the YOLOv11x-seg model based on the Group I test dataset. The test dataset included 100 impacted maxillary canine observations.

**Figure 9:**
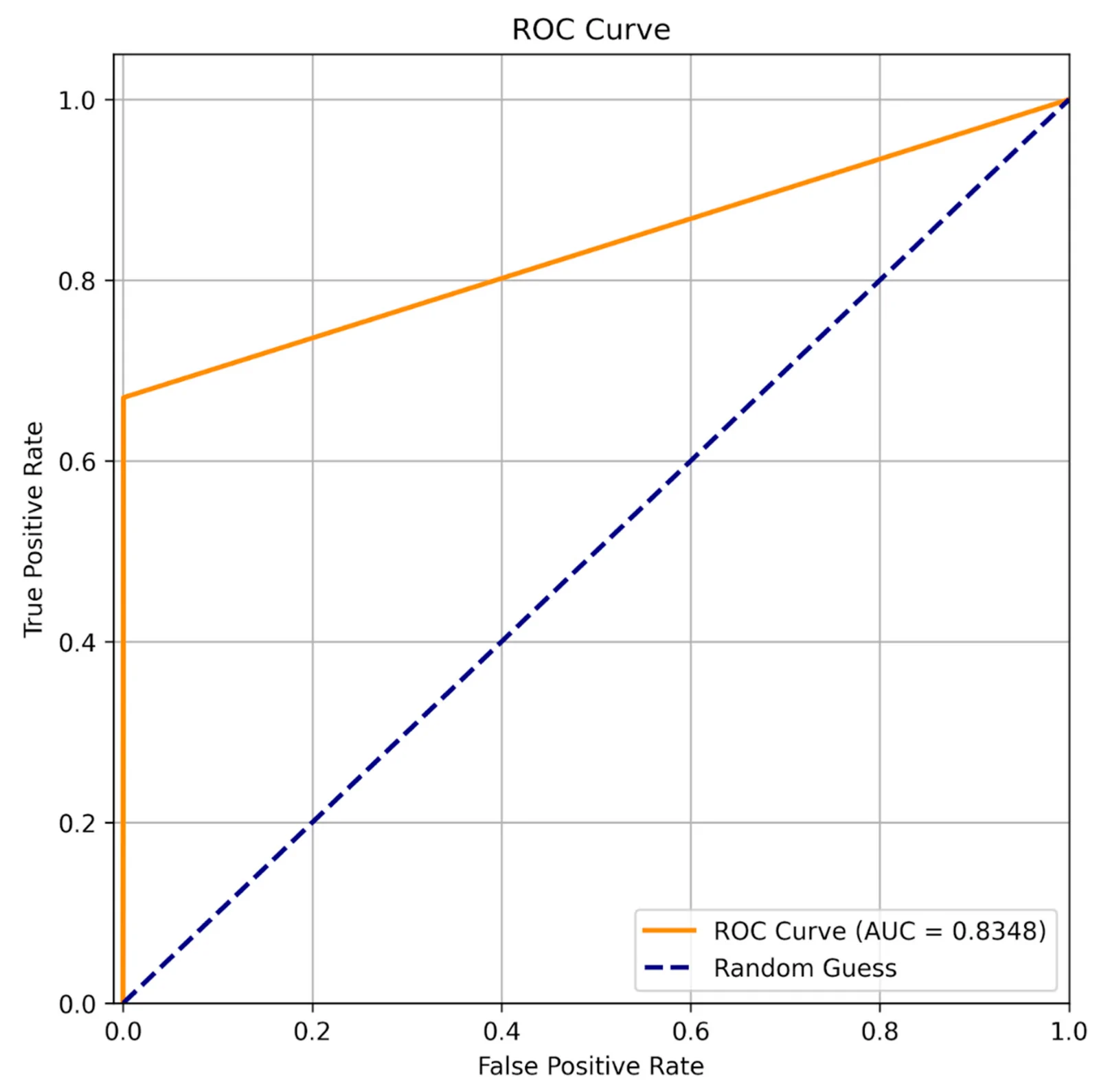
ROC curve and AUC value for the Group I test dataset.

The ROC curve of the YOLOv11x-seg model obtained from the Group I test dataset is presented in Figure 9. The ROC curve illustrates the relationship between the true positive rate (TPR) and the false positive rate (FPR). The area under the curve (AUC) was calculated as 0.8348.

### 3.2. Results Obtained from the Group II Test Dataset

The performance metrics obtained for the positional classification of impacted maxillary canines in the Group II test dataset are presented in Table 2. For buccally impacted canines, the model achieved a precision of 0.48, a recall of 0.76, and an F1 score of 0.59. In the mid-alveolar position, the precision, recall, and F1 score were 0.54, 0.66, and 0.59, respectively. For palatally impacted canines, the model demonstrated the highest performance, with a precision of 0.78, a recall of 0.85, and an F1 score of 0.81.

The critical success index, TP/(TP + FP + FN), of the model was 0.51 (95% CI, 0.422–0.603), while the mean Average Precision values were calculated as mAP at 0.5 of 0.80 and mAP at 0.5 to 0.95 of 0.67. The average Dice coefficient, reflecting segmentation performance, was 0.70, and the Jaccard or Intersection over Union value was determined to be 0.65.

**Figure 10:**
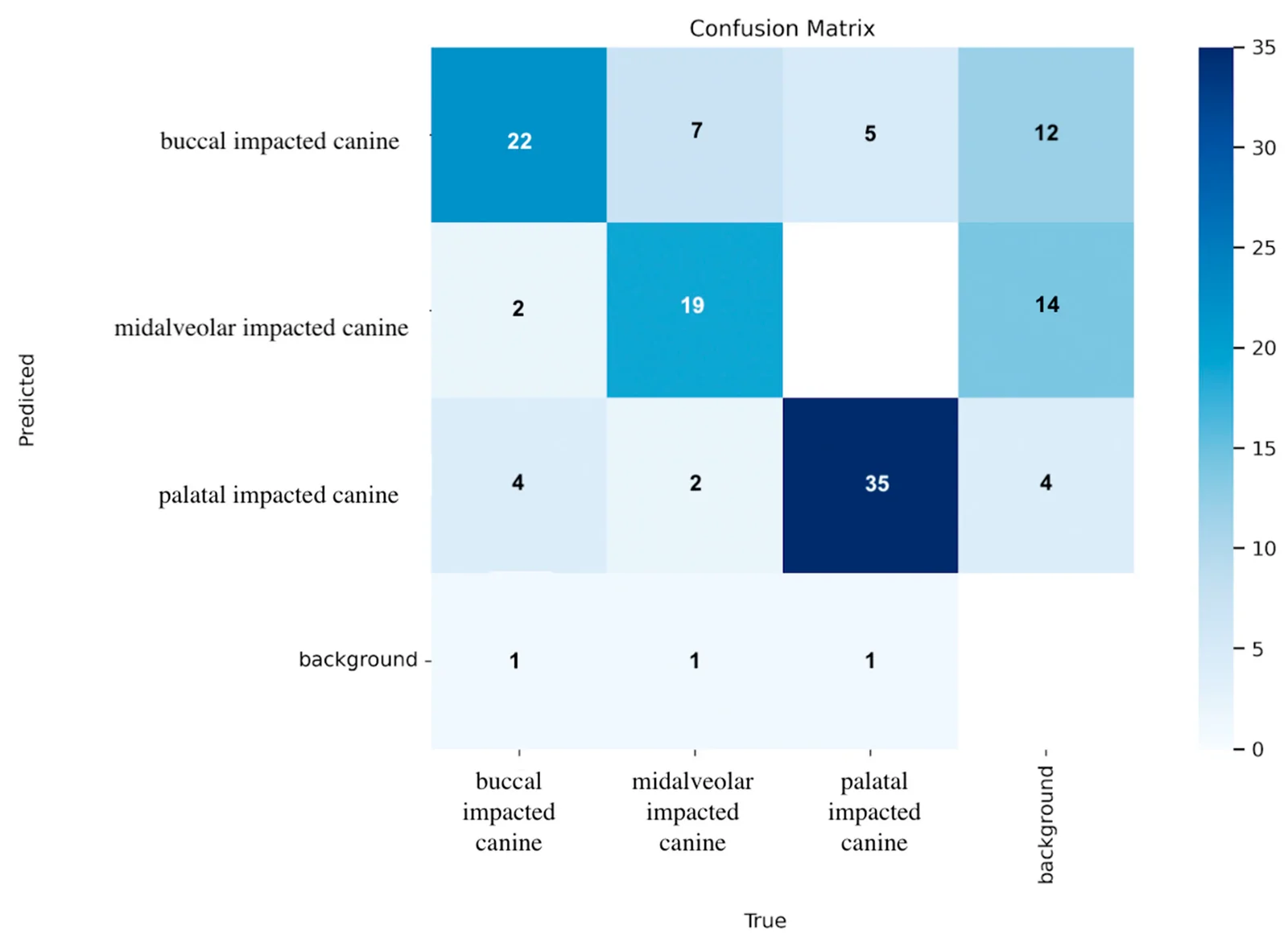
Confusion matrix for the Group II test dataset.

Figure 10 illustrates the confusion matrix of the YOLOv11x-seg model based on the Group II test dataset. The test dataset included 29 buccal, 29 mid-alveolar, and 41 palatal impacted canine observations. The confusion matrix demonstrated that correct classification was highest for the palatal position. Misclassifications were primarily observed between buccal and mid-alveolar positions, with additional confusion involving the buccal and palatal classes.

**Figure 11:**
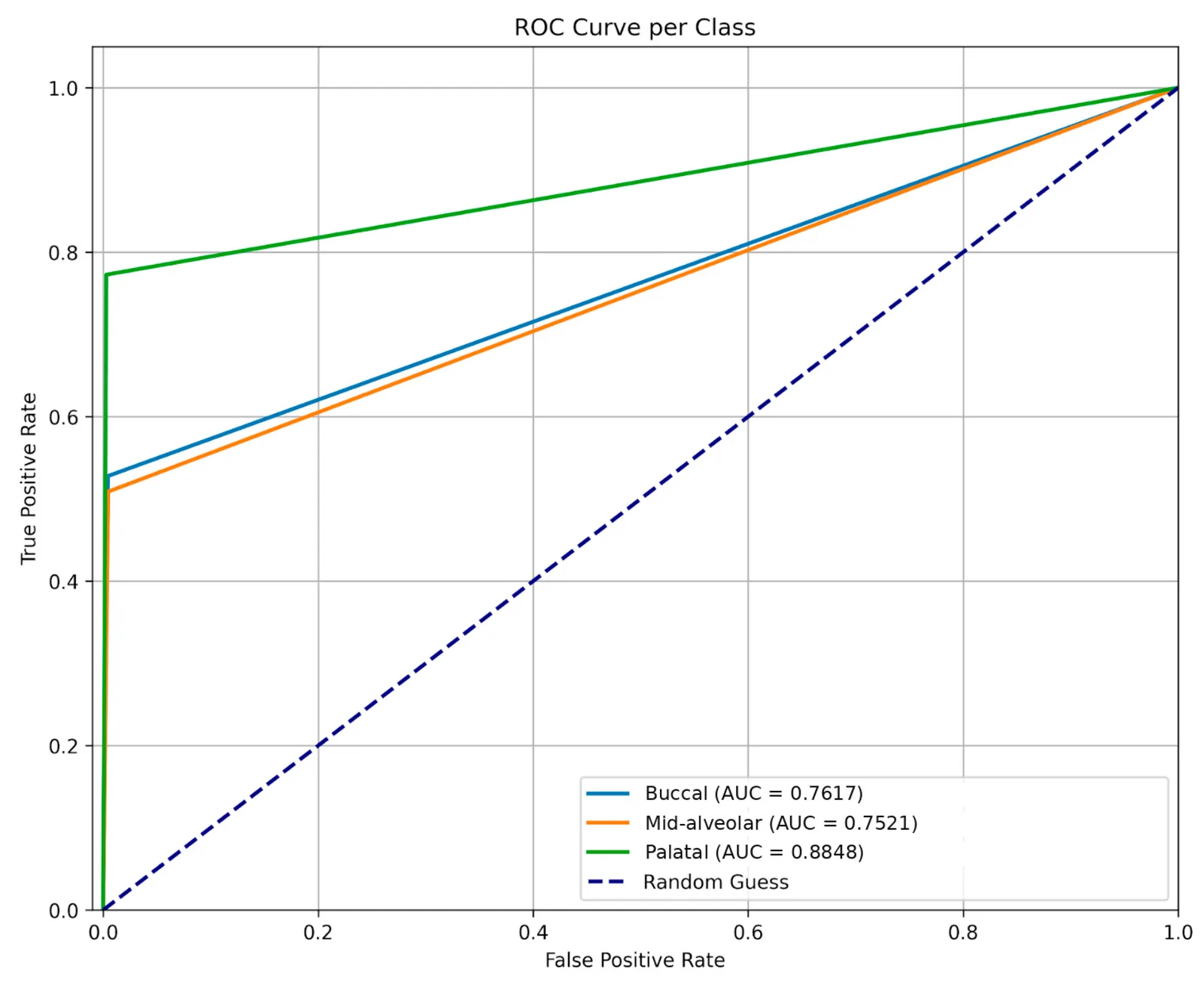
ROC curves and AUC values for the Group II test dataset.

The ROC curves of the YOLOv11x-seg model obtained from the Group II test dataset are presented in Figure 11. The AUC values were calculated as 0.7617 for the buccal position, 0.7521 for the mid-alveolar position, and 0.8848 for the palatal position. The highest AUC value was observed for the palatal position, whereas the lowest value was observed for the mid-alveolar position.

### 3.3. Results Obtained from the Group III Test Dataset

The performance metrics obtained for root resorption detection in the Group III test dataset are presented in Table 3. Among the nine teeth with root resorption in the test dataset, only one was correctly identified by the model (TP = 1). Accordingly, for teeth with root resorption, the model achieved a precision of 0.25, a recall of 0.11, and an F1 score of 0.15. In contrast, for teeth without root resorption, the precision, recall, and F1 score were 0.63, 0.88, and 0.73, respectively.

The critical success index of the model was 0.55 (95% CI, 0.471–0.620), while the mean Average Precision values were calculated as mAP at 0.5 of 0.60 and mAP at 0.5 to 0.95 of 0.32. The Dice coefficient, reflecting segmentation performance, was 0.57, and the Jaccard or Intersection over Union value was determined to be 0.49.

**Figure 12:**
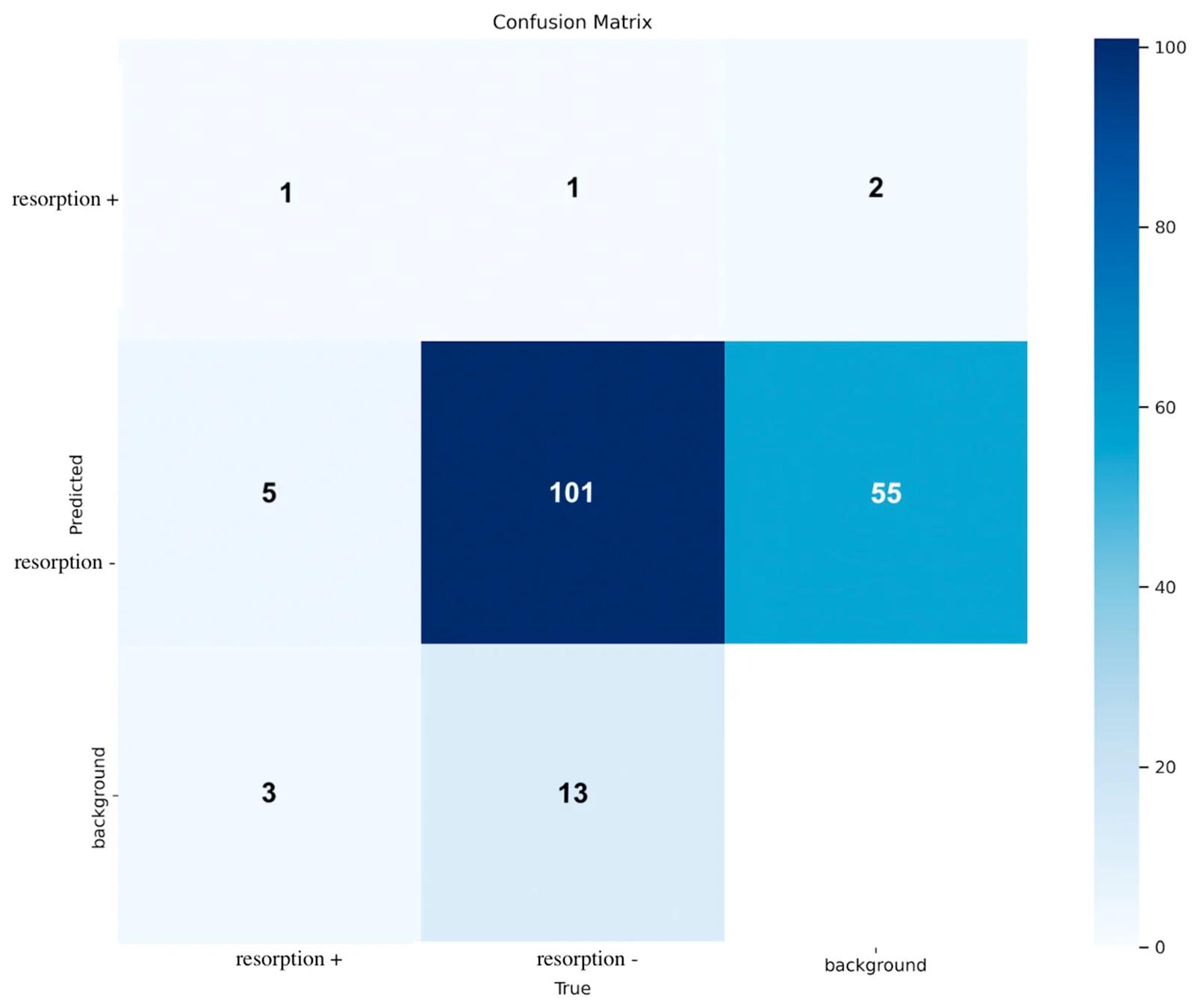
Confusion matrix for the Group III test dataset.

Figure 12 illustrates the confusion matrix of the deep learning model based on the Group III test dataset. The test dataset included 9 resorption-present and 115 resorption-absent observations.

**Figure 13:**
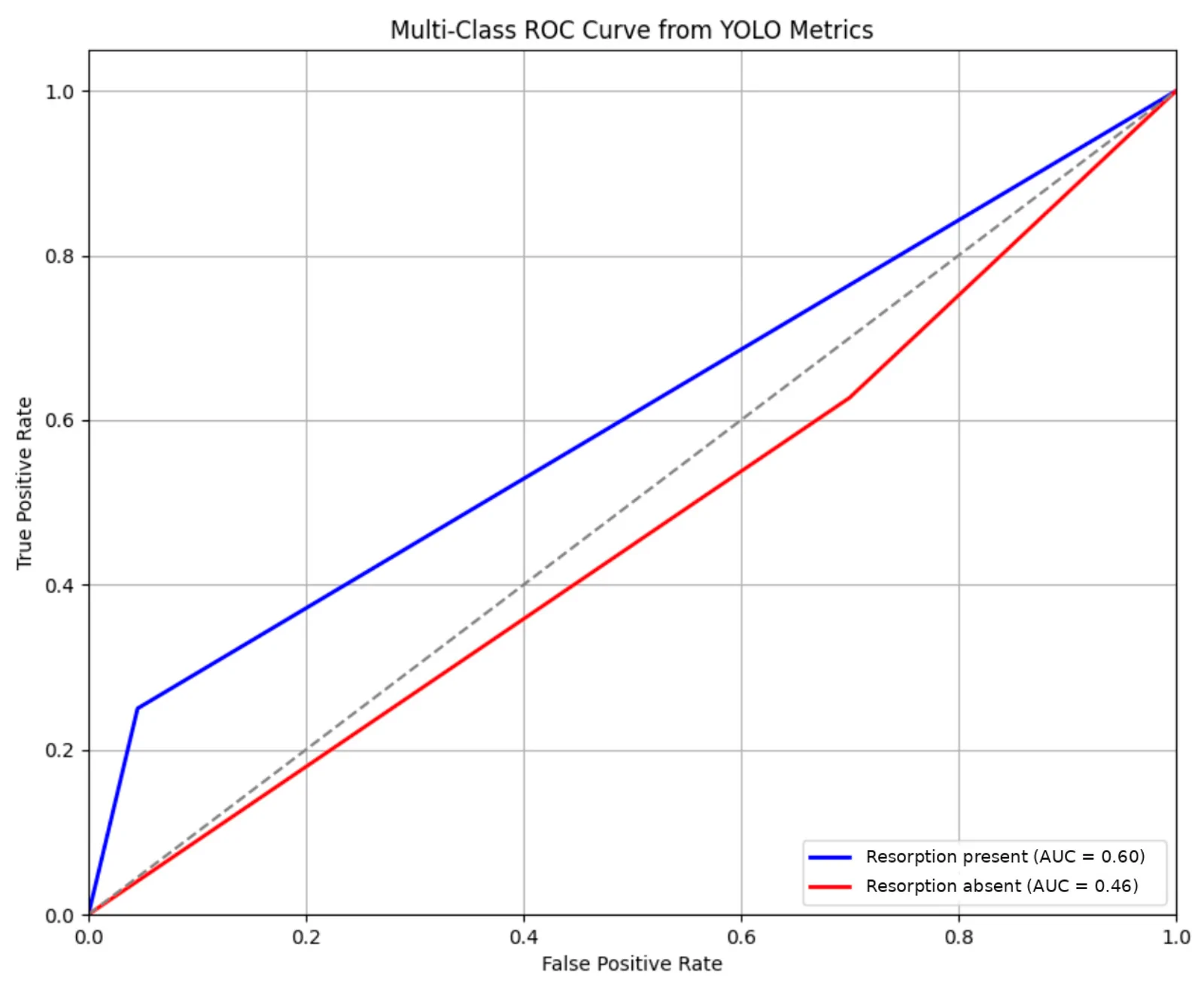
ROC curves and AUC values for the Group III test dataset.

The ROC curves of the YOLOv11x-seg model obtained from the Group III test dataset are presented in Figure 13. The AUC values were calculated as 0.60 for the “resorption present” class and 0.46 for the “resorption absent” class.

### 3.4. Results Obtained from the Group IV Test Dataset

The performance metrics obtained for gamma angle classification in the Group IV test dataset are presented in Table 4. For cases with gamma angles above 65°, the model achieved a precision of 0.57, a recall of 0.91, and an F1 score of 0.70. For cases with gamma angles below 65°, the precision, recall, and F1 score were 0.71, 0.84, and 0.77, respectively.

The critical success index of the model was 0.59 (95% CI, 0.485–0.705), while the mean Average Precision values were calculated as mAP at 0.5 of 0.90 and mAP at 0.5 to 0.95 of 0.73. The Dice coefficient, reflecting segmentation performance, was 0.71, and the Jaccard or Intersection over Union value was determined to be 0.66. For the manual gamma angle measurements performed on panoramic radiographs, the ICC values (two-way model, absolute agreement, single measures) were 0.998 (95% CI, 0.997–0.999) for intraobserver reliability and 0.986 (95% CI, 0.970–0.994) for interobserver reliability. The standard error of measurement, calculated using Dahlberg’s formula from these repeated measurements, was 0.70° for the intraobserver and 1.82° for the interobserver measurements.

**Figure 14:**
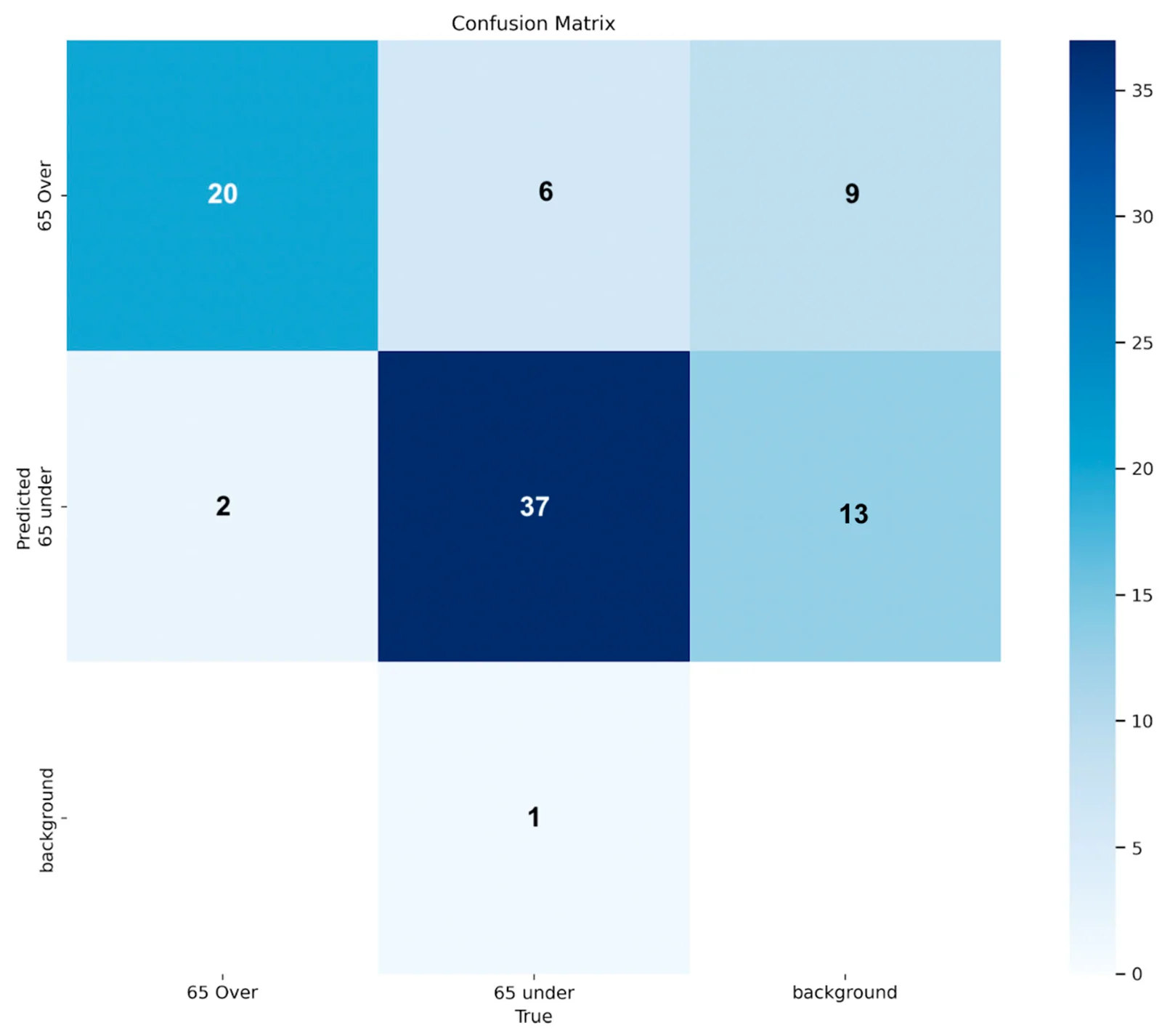
Confusion matrix for the Group IV test dataset.

Figure 14 illustrates the confusion matrix of the YOLOv11x-seg model based on the Group IV test dataset. The test dataset included 22 observations with a gamma angle greater than 65° and 44 observations with a gamma angle below 65°.

**Figure 15:**
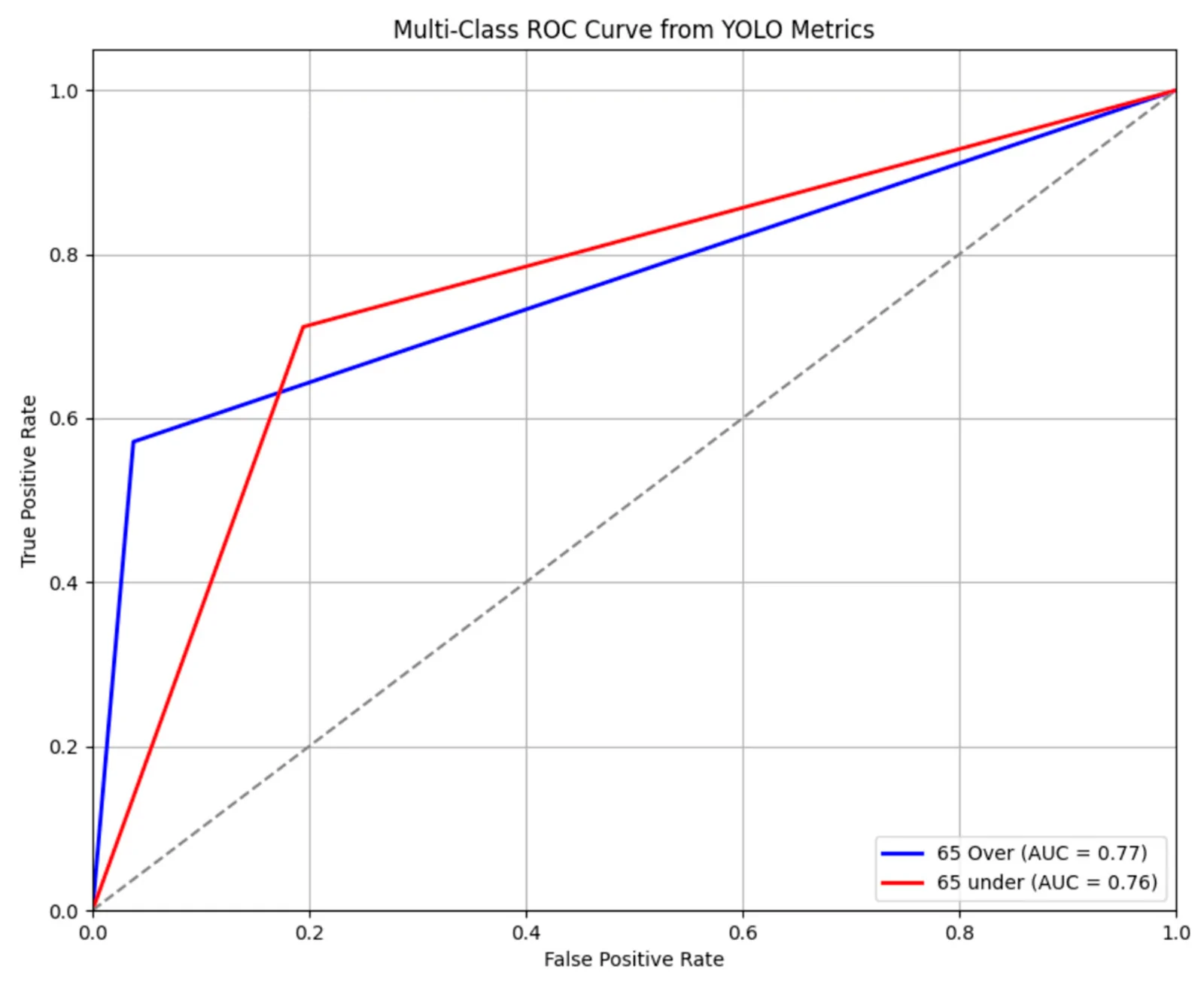
ROC curves and AUC values for the Group IV test dataset.

The ROC curves of the YOLOv11x-seg model obtained from the Group IV test dataset are presented in Figure 15. The AUC values were calculated as 0.76 for the <65° class and 0.77 for the >65° class.

## 4. Discussion

The aim of this study was to evaluate impacted maxillary canines using deep learning-based artificial intelligence models applied to data obtained from panoramic radiographs. Four different analysis groups were examined. CBCT images were used as the reference standard for the assessment of impacted canine position and adjacent root resorption, whereas gamma angle measurements were based on panoramic radiographs.

In the Group I analyses conducted to detect impacted maxillary canines on panoramic radiographs using the YOLOv11x-seg model, high detection performance was observed. The precision of 0.97, recall of 0.98, and F1 score of 0.98 indicate that the model was able to identify impacted canines with high accuracy and balanced performance. In addition, a Dice coefficient of 0.91 and an Intersection over Union value of 0.85 indicate that the model successfully delineated the anatomical contours of impacted canines with high spatial accuracy. However, the high performance observed in Group I should be interpreted in the context of the study design. All radiographs in this group were obtained from patients with at least one impacted maxillary canine, and truly negative panoramic radiographs were not included. Therefore, the dataset represents a spectrum-enriched population, and the reported performance reflects object detection and localization within this preselected population rather than screening performance in an unselected clinical population. Performance may be lower when the model is applied to real-world datasets containing both impacted and non-impacted cases.

Several studies in the literature have investigated the automatic detection of impacted teeth on panoramic radiographs using different deep learning approaches. Başaran et al. [34] reported an F1 score of 0.86 for impacted tooth detection using the Faster R-CNN Inception v2 COCO architecture. Zhicheng et al. [35] employed the Segment Anything Model based MedSAM framework and achieved an accuracy of 86.73 percent for impacted tooth detection; however, they reported a relatively low F1 score of 0.5350. Küçük et al. [36] reported an F1 score of 96 percent using a hybrid CNN Transformer model. Çelik [37] compared different architectures for the detection of impacted mandibular third molars on panoramic radiographs and reported that the YOLOv3 model demonstrated the highest performance. Deepa et al. [38] used Fast R-CNN for the automatic detection of impacted canines and reported an accuracy of 98.3 percent, a recall of 96.5 percent, and a precision of 96.8 percent; however, no F1 score was reported. Tokatlı et al. [39] compared four models for the automatic classification of impacted maxillary canines and reported the highest performance using the VGG16 architecture.

Existing studies demonstrate that impacted teeth can be detected on panoramic radiographs with high accuracy using deep learning models, and detection performance has improved substantially with ongoing advancements in deep learning architectures and the availability of larger and more diverse datasets [34,35,36,37,38,39]. However, it should be noted that the identification of impacted maxillary canines on panoramic radiographs is generally a relatively straightforward task for experienced orthodontists. Therefore, the clinical value of artificial intelligence in this context may not primarily lie in replacing expert evaluation, but rather in supporting specific aspects of clinical workflow. In this regard, the high detection performance observed within the selected study population highlights the potential of the model as a supportive tool rather than a standalone diagnostic solution.

In addition to these findings, the use of cone beam computed tomography data as the reference standard in the present study provides a more reliable basis for confirming the presence and position of impacted teeth. In this respect, the current study differs from previous investigations based solely on panoramic radiographs and makes an important contribution by enabling an objective evaluation of the accuracy of deep learning-based models.

The literature reveals that deep learning-based studies focusing on the positional classification of impacted maxillary canines on panoramic radiographs remain limited. Existing research has predominantly concentrated on general diagnostic tasks such as impacted tooth detection, segmentation, or tooth numbering. In the present study, in addition to the automatic detection of impacted maxillary canines, the classification of their positions as buccal, mid-alveolar, and palatal was also targeted.

In the Group II analyses performed to determine the position of impacted maxillary canines on panoramic radiographs, the YOLOv11x-seg model demonstrated position-dependent performance variability. Among the three classes, the highest performance was achieved in the palatal position, with an F1 score of 0.81, a precision of 0.78, and a recall of 0.85. These findings indicate that the model was more effective in accurately identifying palatally positioned canines while minimizing missed detections. This result is further supported by the highest area under the ROC curve value of 0.8848 observed for the palatal class.

Lower performance was observed for the mid-alveolar and buccal positions, both with an F1 score of 0.59. In the mid-alveolar class, the precision was 0.54 and the recall was 0.66, whereas in the buccal class, the precision was 0.48 and the recall was 0.76. The similar anatomical projections of buccal and mid-alveolar positions on panoramic radiographs are considered to reduce the discriminative capacity of two-dimensional imaging, thereby limiting the ability to distinguish between these two positions. Analysis of the confusion matrix showed that errors resulted from both inter-class misclassification and confusion with the background (Figure 10). Background false-positive detections represented an important source of error, with 12 buccal, 14 mid-alveolar, and 4 palatal predictions occurring in regions corresponding to background. The low precision of the buccal class resulted from the fact that only 22 of 46 buccal predictions were correct; the remaining buccal predictions consisted of misclassified mid-alveolar and palatal canines and false-positive detections in background regions.

Indeed, several studies have highlighted the inherent limitations of two-dimensional panoramic radiography in accurately localizing impacted maxillary canines due to factors such as image distortion, magnification, and superimposition of anatomical structures [3,40]. These diagnostic discrepancies align with previous investigations showing that two-dimensional imaging frequently fails to determine the true labiopalatal location of impacted canines when verified against in vitro (dry skull) or in vivo (surgical) gold standards, whereas CBCT provides higher diagnostic accuracy [14,15].

The critical success index of 0.51 further indicates that the model demonstrated limited overall performance in positional classification. Although the model requires further improvement in differentiating buccal and mid-alveolar canines, the high performance achieved in the palatal position suggests that palatal impactions may be more readily identifiable from panoramic morphology, whereas buccal and mid-alveolar localization remains unreliable. In the present study, class balance was achieved by using equally sized groups consisting of 325 cases for each tooth position. In contrast, Minhas et al. [41] aimed to identify the positions of impacted maxillary canines on panoramic radiographs using a Generative Adversarial Network based deep learning approach in a study including 123 patients. They reported an overall accuracy of 41 percent for positional detection of impacted maxillary canines, with accuracy rates of 64 percent for the buccal position, 33 percent for the mid-alveolar position, and 12 percent for the palatal position. This marked performance difference is considered to be attributable to the limited sample size and the imbalanced class distribution in the dataset used by Minhas et al. [41], which constrained the learning capacity of the model. In addition, the authors [41] reported loss of detail and blurring in reconstructed images, particularly noting reduced clarity at the tooth–bone boundaries.

Salmanpour and Akpınar [42] evaluated the performance of ChatGPT 4.0 in determining the labio-palatal position of impacted canines in a study involving 105 patients. In their study, ChatGPT 4.0 demonstrated an overall accuracy of 37.1 percent for positional determination. Precision and recall values were reported as 48.4 percent and 61.2 percent for the palatal position, 21.7 percent and 19.2 percent for the mid-alveolar position, and 20 percent and 13.3 percent for the labial position. Consistent with the present study, the highest performance in that study was also observed for palatally impacted canines, whereas the mid-alveolar and labial positions were poorly differentiated. The reliance of ChatGPT on text-based inference rather than direct image analysis is considered to have resulted in limited performance in clinical image interpretation tasks.

In the Group III analyses evaluating root resorption in teeth adjacent to impacted maxillary canines, only one of the nine teeth with root resorption in the test dataset was correctly identified by the model (TP = 1). The F1 score was 0.15 for the “resorption present” class, whereas an F1 score of 0.73 for the “resorption absent” class indicated that the model classified non-resorptive cases more successfully. Given the small number of resorption-positive cases and the presence of only one true-positive detection, the performance of the model for the “resorption present” class could not be reliably estimated from the present test dataset. Nevertheless, with a recall of 0.11, the current model is inadequate and clinically unreliable for the detection of root resorption on panoramic radiographs. Given the marked class imbalance in Group III, the critical success index should also be interpreted cautiously and together with the class-specific performance metrics. Because 93.1% of the adjacent teeth were resorption-negative, a no-information classifier labelling all teeth as resorption-absent would reach an apparent conventional accuracy of approximately 93%; this value is not directly comparable with the critical success index, which does not include true negatives, and it illustrates why overall measures are misleading for this task. Larger datasets containing sufficient numbers of resorption-positive cases are required to more reliably assess and improve model performance.

The limited performance in root resorption detection may reflect not only model-related factors but also the inherent information limitations of panoramic radiography. Importantly, artificial intelligence cannot recover three-dimensional diagnostic information that is not adequately represented in the two-dimensional panoramic image. Root resorptions on panoramic radiographs are often small lesions with indistinct borders and may be masked by the superimposition of anatomical structures. In particular, early or minor resorptive changes may not be reliably detected on two-dimensional images, especially in regions such as the lateral incisors where overlapping structures are common. These findings are consistent with previous reports indicating that panoramic radiography has limited sensitivity for detecting root resorption and that cone-beam computed tomography remains the reference standard for accurate diagnosis when resorption is suspected [40,43]. Therefore, the model should not be considered a standalone diagnostic tool for resorption detection, and its low sensitivity indicates a potential risk of false reassurance, particularly in cases where early resorptive changes may be overlooked.

The literature includes a limited number of studies addressing root resorption detection on panoramic radiographs. Salmanpour and Akpınar [42] evaluated the performance of ChatGPT 4.0 in detecting root resorption in adjacent incisors on panoramic radiographs in a study involving 50 patients and reported an accuracy of 46.0 percent for resorption detection. Pirayesh et al. [44] drew attention to the low AUC values previously reported for panoramic radiographs in the diagnosis of canine-induced root resorption and evaluated the effectiveness of deep learning models in diagnosing impacted canine-related root resorption using cone beam computed tomography images from 50 patients. In their study comparing five different models, the highest performing model achieved an F1 score of 0.62.

In Group IV, the angle formed between impacted maxillary canines and the occlusal plane was evaluated. In the present study, the threshold value of 65° reported by Katsnelson et al. [28] was adopted as a reference, and the learnability of angular values below and above 65° by deep learning algorithms was examined. In this way, the ability of the model to discriminate between these angular differences was assessed. The model achieved a precision of 0.57, a recall of 0.91, and an F1 score of 0.70 for cases above 65°, and a precision of 0.71, a recall of 0.84, and an F1 score of 0.77 for cases below 65°, indicating that the deep learning approach was capable of learning and distinguishing this complex variable.

The 65° threshold used in the present study represents a clinically meaningful parameter for predicting the buccal or palatal position of impacted maxillary canines. Indeed, previous studies have demonstrated that angulations greater than 65° on panoramic radiographs are strongly associated with buccal impaction, significantly increasing the likelihood of a buccal position [28]. In this context, the ability of the deep learning model to classify the gamma angle as above or below 65° using panoramic radiographs may provide indirect yet clinically valuable information regarding the three-dimensional position of the impacted tooth. This information may assist in determining the most appropriate surgical approach (buccal or palatal) for exposure, as well as in planning orthodontic traction mechanics. Furthermore, a reduced gamma angle may indicate a less favorable tooth inclination, suggesting that initial uprighting of the impacted canine may be required during orthodontic traction. This, in turn, may influence the direction, magnitude, and biomechanical strategy of the applied forces. Angular variations may therefore have clinical relevance not only for positional prediction but also for treatment difficulty and biomechanical planning. In this context, AI-based classification of the gamma angle may improve workflow efficiency by reducing the time required for manual measurements and minimizing operator-dependent variability, thereby providing more consistent and reproducible assessments.

A review of the existing literature reveals that studies focusing on the automatic classification of dental angular positions on panoramic radiographs using deep learning algorithms are very limited. Previous investigations have primarily focused on predicting the angulation or eruption direction of mandibular third molars [45,46], and no studies were identified that addressed the automatic classification of gamma angles of maxillary canines using panoramic radiographs.

Therefore, the potential clinical role of AI-based analysis should be considered as providing additional support during the initial assessment of impacted maxillary canines rather than replacing CBCT when three-dimensional evaluation is clinically indicated. The present study has several limitations. First, the two-dimensional nature of panoramic radiography, as discussed above, may limit the discriminative capability of the models. In Group IV, the ground-truth labels were derived from manual gamma angle measurements on panoramic radiographs rather than CBCT. Therefore, the model performs binary classification based on a two-dimensional measurement and may inherently reflect the distortion and magnification limitations associated with panoramic imaging. Furthermore, all panoramic radiographs were obtained at a single institution using the same imaging system. This may limit the external validity and generalizability of the models, as differences in patient populations, imaging devices, detector characteristics, image-processing algorithms, contrast, patient positioning, field of view, and acquisition protocols across institutions may affect model performance. Future studies should externally validate the models using datasets obtained from different institutions, patient populations, and imaging systems. Because inclusion required both panoramic radiographs and CBCT examinations, the study population represents a CBCT-selected clinical sample, which may introduce spectrum bias and limit the generalizability of the findings. Moreover, no direct comparison with clinician performance was made, which limits the assessment of the added clinical value of the proposed model. Although equally sized groups were created for positional classification, the balanced 1:1:1 distribution does not reflect the natural prevalence of buccal, mid-alveolar, and palatal impactions in clinical practice. Because positive and negative predictive values are influenced by prevalence, predictive values derived from this balanced dataset may differ from those observed in routine clinical populations and should not be directly generalized to clinical practice. Increasing the overall dataset size has the potential to further improve model performance. The limited number of cases in the resorption present group represents another constraint that may have negatively affected model performance in this subgroup. Furthermore, the evaluation of root resorption was limited to a binary classification as present or absent, without grading severity, which prevented assessment of the model’s potential to distinguish different degrees of resorption. Future studies may address this limitation by evaluating root resorption according to different severity levels. The limited number of atypical canine cases in the dataset may also restrict the evaluation of model performance in such conditions. In addition, Group I did not include an independent cohort of patients without impacted maxillary canines; therefore, its results reflect object detection and localization performance rather than patient-level diagnostic screening performance. Finally, k-fold cross-validation or repeated random splitting was not performed; therefore, the findings are based on a single patient-level data split.

## 5. Conclusions

Four independently trained YOLOv11x-seg models were evaluated for the automatic assessment of impacted maxillary canines on panoramic radiographs. The models showed promising performance for the detection and segmentation of impacted canines within the selected study population; however, their performance was limited for positional assessment, moderate for gamma angle classification, and inadequate for the detection of root resorption, for which the small number of resorption-positive cases also prevented a reliable estimation of performance. These models may serve as a supportive tool during the initial assessment but should not replace CBCT when three-dimensional evaluation is indicated. Larger, multicenter datasets, including patients without impacted canines and sufficient resorption-positive cases, are required before clinical application.

## Figures and Tables

**Table 1:** Performance of the YOLOv11x-seg model on the Group I test dataset.

	Critical Success Index (95% CI)	Precision (95% CI)	Recall (95% CI)	F1 Score (95% CI)
Impacted canine	0.95 (0.903–0.990)	0.97 (0.916–0.994)	0.98 (0.930–0.998)	0.98 (0.949–0.995)

**Table 2:** Performance of the YOLOv11x-seg model on the Group II test dataset.

Position	Precision (95% CI)	Recall (95% CI)	F1 Score (95% CI)
Buccal	0.48 (0.329–0.631)	0.76 (0.565–0.897)	0.59 (0.441–0.710)
Mid alveolar	0.54 (0.366–0.712)	0.66 (0.457–0.821)	0.59 (0.436–0.725)
Palatal	0.78 (0.629–0.888)	0.85 (0.708–0.944)	0.81 (0.714–0.895)
Macro average	0.60 (0.515–0.683)	0.76 (0.667–0.840)	0.66 (0.579–0.741)
Micro average	0.60 (0.516–0.688)	0.77 (0.683–0.848)	0.68 (0.594–0.752)

**Table 3:** Performance of the YOLOv11x-seg model on the Group III test dataset.

Class	Precision (95% CI)	Recall (95% CI)	F1 Score (95% CI)
Resorption present	0.25 (0.006–0.806)	0.11 (0.003–0.482)	0.15 (0.000–0.444)
Resorption absent	0.63 (0.548–0.702)	0.88 (0.804–0.932)	0.73 (0.669–0.788)

**Table 4:** Performance of the YOLOv11x-seg model on the Group IV test dataset.

Gamma Angle Category	Precision (95% CI)	Recall (95% CI)	F1 Score (95% CI)
Below 65°	0.71 (0.569–0.829)	0.84 (0.699–0.934)	0.77 (0.667–0.857)
Above 65°	0.57 (0.394–0.737)	0.91 (0.708–0.989)	0.70 (0.545–0.825)

## Data Availability

The data presented in this study are available on request from the corresponding author. The model weights and code are not publicly available because they are part of the technical infrastructure of the external artificial intelligence company involved in the technical development of the models.

## Supplementary Materials

The following supporting information can be downloaded at: https://www.mdpi.com/article/10.3390/diagnostics16193167/s1. Figure S1: Training and validation curves of the four independently trained YOLOv11x-seg models: (A) Group I, (B) Group II, (C) Group III, and (D) Group IV. Loss components (box, segmentation, classification, and distribution focal loss) and validation metrics for bounding boxes (B) and masks (M) are shown per epoch. Training was terminated by early stopping (patience, 50 epochs). In Groups I and II, the validation segmentation loss increased abruptly and the mask metrics decreased late in training (from epoch 107 in Group I and after approximately epoch 190 in Group II); the final models correspond to the best validation checkpoints obtained before this point (epoch 85 in Group I, with training stopped at epoch 135). In Group IV, the best validation checkpoint was obtained at epoch 71, and training stopped at epoch 121.

## Abbreviations

The following abbreviations are used in this manuscript:

AI	Artificial Intelligence
AP	Average Precision
AUC	Area Under the Curve
CBCT	Cone-Beam Computed Tomography
CNN	Convolutional Neural Network
DICOM	Digital Imaging and Communications in Medicine
IoU	Intersection over Union
mAP	Mean Average Precision
MedSAM	Medical Segment Anything Model
R-CNN	Region-Based Convolutional Neural Network
ROC	Receiver Operating Characteristic
YOLO	You Only Look Once
