# Supplementary material for: Evaluation of the Position of Impacted Maxillary Canines on Panoramic Radiographs Using Deep Learning with CBCT as the Reference Standard
Source: Diagnostics (Basel). 2026 Sep 29;16(19):3167. doi: 10.3390/diagnostics16193167 (PMC13663945; doi:10.3390/diagnostics16193167)
Supplement: Supplementary file 1 [file diagnostics-16-03167-s001.zip › diagnostics-4542324-supplementary.pdf]

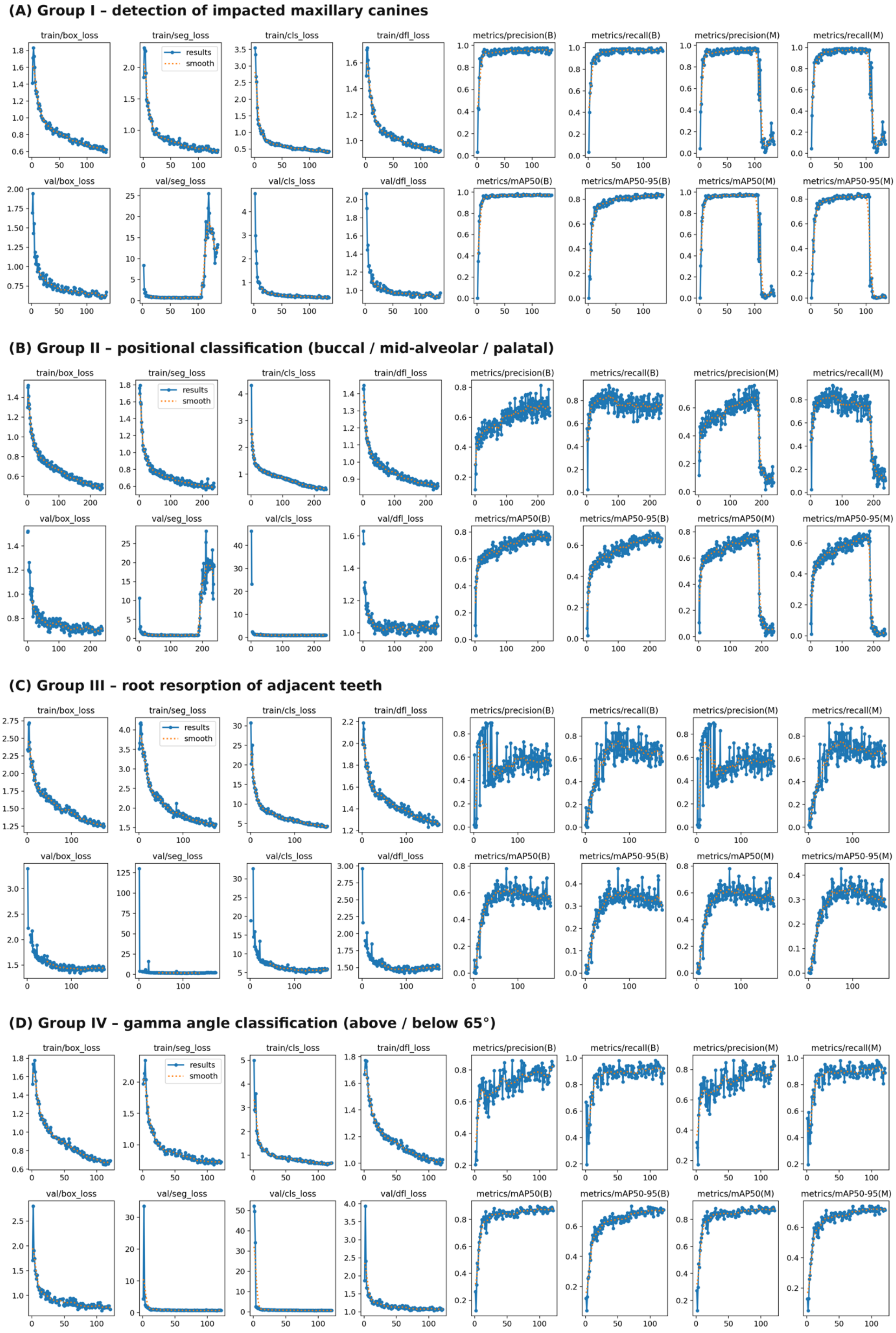
(A) Group I – detection of impacted maxillary canines
train/box_loss
train/seg_loss
train/cls_loss
train/dfl_loss
metrics/precision(B)
metrics/recall(B)
metrics/precision(M)
metrics/recall(M)
val/box_loss
val/seg_loss
val/cls_loss
val/dfl_loss
metrics/mAP50(B)
metrics/mAP50-95(B)
metrics/mAP50(M)
metrics/mAP50-95(M)
results
smooth
(B) Group II – positional classification (buccal / mid-alveolar / palatal)
train/box_loss
train/seg_loss
train/cls_loss
train/dfl_loss
metrics/precision(B)
metrics/recall(B)
metrics/precision(M)
metrics/recall(M)
val/box_loss
val/seg_loss
val/cls_loss
val/dfl_loss
metrics/mAP50(B)
metrics/mAP50-95(B)
metrics/mAP50(M)
metrics/mAP50-95(M)
results
smooth
(C) Group III – root resorption of adjacent teeth
train/box_loss
train/seg_loss
train/cls_loss
train/dfl_loss
metrics/precision(B)
metrics/recall(B)
metrics/precision(M)
metrics/recall(M)
val/box_loss
val/seg_loss
val/cls_loss
val/dfl_loss
metrics/mAP50(B)
metrics/mAP50-95(B)
metrics/mAP50(M)
metrics/mAP50-95(M)
results
smooth
(D) Group IV – gamma angle classification (above / below 65°)
train/box_loss
train/seg_loss
train/cls_loss
train/dfl_loss
metrics/precision(B)
metrics/recall(B)
metrics/precision(M)
metrics/recall(M)
val/box_loss
val/seg_loss
val/cls_loss
val/dfl_loss
metrics/mAP50(B)
metrics/mAP50-95(B)
metrics/mAP50(M)
metrics/mAP50-95(M)
results
smooth

Supplementary Materials: Figure S1. Training and validation curves of the four independently trained YOLOv11x-seg models: (A) Group I, (B) Group II, (C) Group III, and (D) Group IV. Loss components (box, segmentation, classification, and distribution focal loss) and validation metrics for bounding boxes (B) and masks (M) are shown per epoch. Training was terminated by early stopping (patience, 50 epochs). In Groups I and II, the validation segmentation loss increased abruptly and the mask metrics de-creased late in training (from epoch 107 in Group I and after approximately epoch 190 in Group II); the final models correspond to the best validation checkpoints obtained before this point (epoch 85 in Group I, with training stopped at epoch 135). In Group IV, the best validation checkpoint was obtained at epoch 71, and training stopped at epoch 121.
